# The impact of price promotions on confectionery and snacks on the energy content of shopping baskets: A randomised controlled trial in an experimental online supermarket

**DOI:** 10.1016/j.appet.2023.106539

**Published:** 2023-07-01

**Authors:** Madison Luick, Rachel Pechey, Georgina Harmer, Lauren Bandy, Susan A. Jebb, Carmen Piernas

**Affiliations:** a Nuffield Department of Primary Care Health Sciences, Radcliffe Observatory Quarter, University of Oxford, Oxford, OX2 6GG, UK; b Department of Biochemistry and Molecular Biology II, Institute of Nutrition and Food Technology, Center for Biomedical Research (CIBM), University of Granada, Spain

**Keywords:** HFSS, Confectionery, Price, Promotion, RCT

## Abstract

Overconsumption of foods high in fat, sugars, and salt (HFSS) poses a significant risk to health. The government in England has passed legislation that would limit some price promotions of HFSS foods within supermarkets, but evidence regarding likely impacts of these policies, especially in online settings, is limited. This study aimed to determine whether there were any differences in the energy and nutrient content of shopping baskets after removing promotions on HFSS foods in an online experimental supermarket. UK adults (n = 511) were asked to select food from four categories with a £10 budget in an online experimental supermarket: confectionery; biscuits and crackers; crisps, nuts and snacking fruit; cakes and tarts. They were randomly allocated to one of two trial arms: (1) promotions present (matched to promotion frequency seen in a major UK retailer) (n = 257), or (2) all promotions removed from all products within the target food categories (n = 254). The primary outcome analysis used linear regression to compare total energy (kcal) of items placed in shopping baskets when promotions were present vs. absent, while secondary analyses investigated differences in nutrients and energy purchased from individual food categories. Mean energy in food selected without promotions was 5156 kcal per basket (SD 1620), compared to 5536 kcal (SD 1819) with promotions, a difference of −552kcal (95%CIs: −866, −238), equivalent to 10%. There were no significant differences in energy purchased for any individual category between groups. No evidence was found of other changes in nutritional composition of baskets or of significant interactions between the impact of promotions and participant characteristics (gender, age, ethnicity) on energy purchased. Removing promotions on HFSS foods resulted in significantly less total energy selected in an online experimental supermarket study.

## 1 Background

In England, in common with most high and middle income countries, we eat more energy, free sugars, saturated fat and salt than recommended for good health (Public Health England, 2018). These nutrients contribute to the burden of major chronic diseases, including cardiovascular disease and diabetes, principally through effects on blood cholesterol, blood pressure, insulin sensitivity or body weight (He et al., 2014; Mozaffarian et al., 2010; Te Morenga et al., 2014). In addition, persistent inequalities in dietary intake underpin inequalities in long-term health outcomes (Maguire & Monsivais, 2015).

Food purchasing is a precursor to food consumption, and a critical target for interventions to improve dietary patterns. Supermarkets account for the majority of the weekly expenditure on food and drinks, estimated to be around 87% of all UK retail grocery sales (Department for Environment Food and Rural Affairs, 2015). Impactful, scalable and sustainable approaches within supermarkets are particularly needed in order to shift population-level intakes to be closer to the recommendations for energy, sugars, saturated fat and salt.

Previous systematic reviews have identified effective strategies to support dietary change in supermarkets (Adam & Jensen, 2016; Cameron et al., 2016; Escaron et al., 2013; Hartmann-Boyce et al., 2018; Shaw, Ntani, Baird, & Vogel, 2020), including economic (e.g. price, promotions) and structural changes (such as increasing availability, more prominent positioning of products, and changing product proximity). Evidence of effectiveness is generally stronger for economic (e.g. price interventions or price promotions), positioning and availability interventions rather than educational interventions alone (Brambila-Macias et al., 2011). However, many of the interventions described in the literature are multi-component, and there is a challenge in attributing their effectiveness to any single strategy, such as price promotions.

In 2017, NHS Health Scotland published a rapid review of previous studies, systematic reviews, and grey literature on the influence of retail environments and promotions, suggesting ways government policy could restrict HFSS promotions to impact purchasing behaviour, such as restricting multi-buys. The review suggests that these types of universal intervention have a greater chance of being applied equitably, and, as such, having a greater benefit for all groups compared to interventions that focus more on individual decisions, such as healthy eating or knowledge campaigns, which have been shown to occasionally widen disparities in food purchasing or consumption (Martin, Bauld, & Angus, 2017). In 2020 the government in England laid out plans to introduce legislation to restrict the promotion of high fat, salt or sugar (HFSS) foods, by restricting volume promotions such as ‘Buy One Get One Free’. This legislation was expected to come into force in October 2022, but has since been postponed by one year (Department of Health and Social, 2022). Estimated effects on UK sales have been modelled based on the impact of removing price promotions on sugar-sweetened beverages in Australian retail settings. This model predicted significant savings from reduced consumption and a corresponding reduction in population health burden (Huse et al., 2020).

Few studies have considered the effectiveness of interventions that may be delivered online. Online shopping has grown rapidly, accelerated by the COVID-19 pandemic. In January and February 2021, online grocery shopping sales in the UK were up over 140% from sales the previous year (Office for National Statistics, 2022b). As the pandemic has progressed, there has been some decline in these sales, but online grocery shopping continues to surpass pre-pandemic levels, with 20% of the population having bought groceries online in the three months before August 2021 and approximately 13% of all grocery sales coming from online sales (McKevitt, 2021). There is some evidence that baseline purchases made online tend to be healthier than purchases in physical stores (Huyghe et al., 2017), and that purchasing decisions in the online environment are less influenced by the microenvironment than in the physical environment (Martin, Bauld, & Angus, 2017). However little is known about the effectiveness of interventions delivered online to change behaviour.

The aim of this study was to test the impact of removing price promotions on foods high in fats, sugars, and salt (HFSS), such as confectionery and snacks, on the energy content of items selected while shopping online in a representative sample of UK adults.

## 2 Methods

### 2.1 Design

This was a randomised controlled trial with a parallel design. Participants were randomised to complete a shopping task in an experimental supermarket platform under one of two conditions: Price promotions removed from confectionery and snack products vs. price promotions present.

The online survey platform Qualtrics automatically randomised participants on a 1:1 basis, achieving allocation concealment with investigators unaware and unable to manipulate study parameters after initial study set up. Participants were only aware of the trial arm that they were exposed to and were unaware of the other trial arm. In addition, the statistician who conducted primary analysis was blinded to intervention allocation.

Ethics approval was granted 29/09/2021 by the Central University Research Ethics Committee, University of Oxford (Ref: R65010/RE006). The study was pre-registered on clinicaltrials.gov (NCT05098223; https://clinicaltrials.gov/ct2/history/NCT05098223).

### 2.2 Intervention

The study used an experimental online supermarket platform, hosted by the University of Oxford, which emulates a real online supermarket for research purposes relating to food purchasing interventions (www.woodssupermarket.co.uk). The site is populated with approximately 23,000 supermarket products that were available to purchase in April 2019, taken from foodDB, a database of food and drinks available for purchase in six UK online supermarkets (Harrington et al., 2019). Participants interact with the site in a similar manner to a real online supermarket, but do not spend money or receive their selected items.

Participants were asked to complete an online shopping task and were randomly allocated to one of the two following groups when shopping online.

1. Promotions removed: No promotions present on any of the products within the target food categories [confectionery; biscuits and crackers; crisps, nuts and snacking fruit; cakes and tarts] offered to participants when searching for products.
2. Promotions included: Participants in this group saw a version of the website which reproduced the types and frequency of promotions that can be found in an online supermarket. Promotions were applied to match the types and depth of promotions on the website of the largest UK retailer that were present in a specific week, shortly before the study launch. For example, temporary price reductions were applied to a matched percentage of food products within the target categories [confectionery (31%); biscuits and crackers (18%); crisps, nuts and snacking fruit (17%); cakes and tarts (15%)]. See Supplementary Table S1 for percentage values.

### 2.3 Participants

There are no previously reported data from similar trials to guide the estimation of the effect size, standard deviation, and sample size. However, a study in Australia implemented a complex intervention within real supermarkets where promotions on high sugar products were removed, including confectionery and sugary drinks (Brimblecombe et al., 2020). This study found a total reduction of −22% in sales (g/MJ) in confectionery, a difference of −4.5% (SD 15) compared to control; and a reduction of −8% in sugar-sweetened soft drinks, a difference of −13% compared to control. As such, a total sample of 500 participants (250 in each group) would be needed to detect a minimum effect size of −4.5% in total energy from the target categories, with 90% power, 5% alpha and 10% attrition.

Participants were recruited in October and November 2021 from the volunteer panel Prolific Academic (https://www.prolific.co). A screening questionnaire identified eligible participants – adults resident in the UK fluent in English. People who were following a vegan, gluten-free, dairy-free or sugar-free diet were not eligible to participate to minimise the risk of unbalancing the groups with people choosing from a restricted selection of products. Participants needed access to a computer and the internet. Using a trial screening feature in Prolific, we aimed to recruit participants that were representative of the UK in terms of age, gender and ethnicity. We recruited roughly equal numbers of participants with higher education (degree-level or higher) and those without higher education (A-levels or lower).

### 2.4 Procedure

Following eligibility screening, participants were invited to complete the main shopping task. First, participants completed a baseline questionnaire on demographic characteristics and shopping habits (Appendix B) on Qualtrics. BMI was calculated using heights and weights reported by study participants. Two participants’ BMI were excluded due to reporting unfeasible heights (namely, 1 cm and above 10,000 cm).

They were then randomised to study condition, and redirected to the experimental online supermarket platform, with the explanation that “*This is not a real online supermarket. You will not be asked to spend any of your own money and you will not take home any groceries”.*

During the task, all participants were asked to imagine they were buying snacks for a night in with four friends, and to spend between £5 and £10. They were asked to select products from the following categories, which were named in the way items are presented in the store.

- Confectionery
- Biscuits and crackers
- Crisps, nuts and snacking fruit
- Cakes and tarts

Participants returned to Qualtrics to complete a short post-intervention survey (Appendix B) about the acceptability of the intervention in the online shopping task and perceived influences on their usual shopping behaviour.

Participants were paid 25p for completing the screening survey, and £1.50 for completing the main survey including the shopping task.

### 2.5 Analysis

Baseline differences in demographic characteristics and shopping habits were checked between groups using t-tests and chi-squared tests. For the primary outcome analysis, linear regression models compared total energy (kcal) placed in shopping baskets between the two study groups.

A sensitivity analysis on the primary analysis was conducted including participants who spent at least £5 on products from the target categories (Confectionery; Biscuits and crackers; Crisps, nuts and snacking fruit; Cakes and tarts), to explore the impact for participants who had greater likelihood of being exposed to the promotional activity. As per study instructions, in addition to those that had not spent at least £5 from the target categories, those that spent more than £10 overall were also excluded from the sensitivity analysis.

Secondary outcomes included saturated fat (% energy selected; grams selected; energy (kcal) selected from saturated fat); sugar (% energy selected; grams selected; energy (kcal) selected from sugar); salt (g/100g), that were compared using linear regression models, following the same procedure as for the primary outcome. We also conducted stratified analyses by demographic variables (gender, age group, ethnic group (White vs Non-White), BMI (<30 and ≥ 30 kg/m^2^) groups, education level (A-levels or lower vs. degree or higher), and household income (below £25,000 vs. above £25,000)), and by food category (Confectionery; Biscuits and crackers; Crisps, nuts and snacking fruit; Cakes and tarts), for both primary and secondary outcomes. Data on the Minimum Income Standard was used for the income cut-off, the BMI cut-off was determined by if an individual reported height and weight equating to having obesity or not, and other levels of demographic variables were determined based on population data from the Office for National Statistics in the UK (Davis et al., 2022; Office for National Statistics, 2019; Office for National Statistics, 2020; Office for National Statistics, 2022a). Given the number of comparisons, the threshold for a p-value to be considered significant for secondary analyses was set at 0.003 (Bonferroni adjustment for multiple comparisons).

## 3 Results

**Fig. 1:**
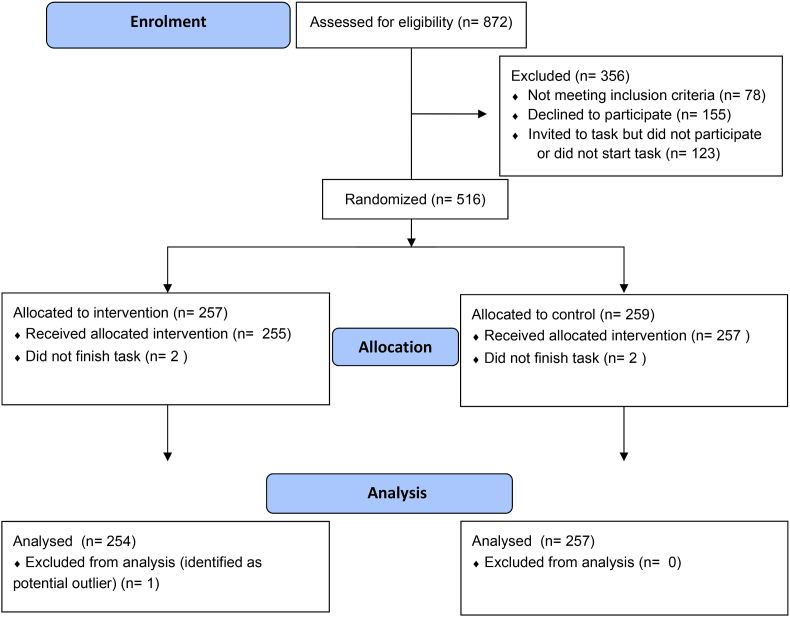
CONSORT flow diagram.

Of the 875 individuals who completed the study screening, 512 completed the shopping task, with 255 in the ‘promotions removed’ group and 257 in the ‘promotions present’ group. The primary analysis was conducted on data from 511 participants (one participant in the promotions group, who purchased only dried herbs, was removed as an outlier) (see CONSORT Flow Diagram, Fig. 1).

Over half of study participants were female, the average participant was aged 39, 26.8% of participants had obesity, and 85.7% of participants had a white background (see Table 1). The participants spent on average about 10.3 min to complete the task and selected an average of 7 items each (min 1- max 23). Weekly grocery spending was found to be slightly imbalanced between groups, so was included in the models as a covariate.

**Table 1:** Characteristics of the trial participants and their selected baskets by study condition. See Supplementary Tables S2 and S3 for further descriptive study population statistics.

		Promotions present (N = 257)	Promotions removed (N = 254)	Total (N = 511)
Gender (N, %)	Male	124 (48.2%)	116 (45.7%)	240 (47.0%)
	Female	131 (50.9%)	138 (54.3%)	269 (52.6%)
Ethnicity (N, %)	Non-white	32 (12.5%)	41 (16.1%)	73 (14.3%)
	White	225 (87.5%)	213 (83.9%)	438 (85.7%)
Education (N, %)	A-levels or lower	131 (51.0%)	135 (53.1%)	266 (52.1%)
	Degree or higher	126 (49.0%)	119 (46.9%)	245 (47.9%)
Household Income (N, %)	Up to £25,000	91 (35.4%)	72 (28.3%)	163 (31.9%)
	Above £25,000	166 (64.6%)	182 (71.7%)	348 (68.1%)
Age Groups (N, %)	18–29	73 (28.4%)	69 (27.2%)	142 (27.8%)
	30–49	125 (48.6%)	128 (50.4%)	253 (49.5%)
	50+	59 (23.0%)	57 (22.4%)	116 (22.7%)
BMI (N, %)	Below 30	181 (70.7%)	173 (68.4%)	354 (69.5%)
	Above 30	75 (29.3%)	80 (31.6.0%)	155 (30.5%)
No of items selected (per participant) (mean, s.d)		7.59 (2.55)	6.44 (2.02)	7.00 (2.38)
Cost (£GBP) (per participant) (mean, s.d)		8.72 (1.24)	9.06 (1.87)	8.89 (1.59)
Weight of products selected (grams) (per participant) (mean, s.d.)		1316 (507)	1228 (397)	1273 (457)
Number of people in household (median, IQR)		3.0 (2.0–4.0)	3.0 (2.0–4.0)	3.0 (2.0–3.0)
Weekly household grocery spending (£) (median, IQR)		63.00 (50.00–100.00)	80.00 (50.00–100.00)	70.00 (50.00–100.00)
Weekly household grocery spending (£) (mean, s.d)		76.36 (44.66)	83.03 (42.55)	79.68 (43.71)

Those in the group with promotions present selected a total of 1951 items, 816 with promotions present (41.8%). Of those items with promotions, 418 (51.2%) had a “half-price” promotion, 227 (27.8%) had a “save one-third” promotion, and 171 (21.0%) had a “save 25%” promotion (See Supplementary Figs. S7–S8). This group placed on average 5536 calories in their baskets, amounting to an average 1317g of product weight, and average cost of £8.72, compared to 5156 calories, 1228g of product weight, and average cost of £9.06 in the group with promotions removed.

When promotions on confectionery and snack foods were removed, participants selected foods containing −552kcal (95%CIs: −866, −238) less compared to when these were present (Table 2). There was no evidence of differences in the proportions of saturated fat, sugar or salt in shopping baskets in the two study conditions. Analyses were also run examining quantity (g and kcal) of saturated fat and sugar, which showed no clear evidence of differences by study condition at p < 0.003 after Bonferroni correction, but were consistent with less saturated fat and sugar (g and kcal) being selected in the promotions removed condition (see Table 2). In the sensitivity analysis, participants selected foods containing −693kcal (95% CI: −1014, −373) less when promotions were removed compared to when they were present, and there was no evidence of differences in the nutrient composition of shopping baskets.

**Table 2:** Comparison of primary and secondary outcomes between trial groups. See Supplementary Table S8 for full regression model output.

	Total Mean (SD)	Promotions present mean (SD) (N = 257)	Promotions removed mean (SD) (N = 254)	Coefficient (95% CI) *(reference: promotions present)*	Coefficient p-value
Energy selected (kcal)	5347(1731)	5536 (1819)	5156 (1620)	−552 (−866, −238)	0.001
Saturated fat (% energy purchased)	14.6% (4.4)	14.5% (4.5)	14.6% (4.3)	0.2 (−0.6, 0.9)	0.671
Saturated fat (kcal)	817 (347)	853 (370)	782 (321)	−71.3 (−131, −11.0)	0.021
Saturated fat (g)	90.8 (38.7)	94.7 (41.1)	86.9 (35.7)	−7.92 (−14.6, −1.22)	0.021
Sugar (% energy purchased)	22.6% (9.4)	22.9% (9.3)	22.3% (9.5)	−0.5 (−2.1, 1.2)	0.574
Sugar (kcal)	1289 (667)	1371 (717)	1206 (603)	−159 (−274, −43.4)	0.007
Sugar (g)	322 (167)	343 (179)	301 (151)	−39.6 (−68.4, −10.9)	0.007
Salt (g/100g)	0.80 (0.29)	0.81 (0.31)	0.79 (0.27)	−0.06 (−0.12, 0.00)	0.065
Energy density (kcal/100g)	464 (127)	470 (127)	457 (126)	−13 (−35, 9)	0.240

*p < 0.003 – the threshold for significance following Bonferroni adjustment for secondary outcomes.

**Fig. 2:**
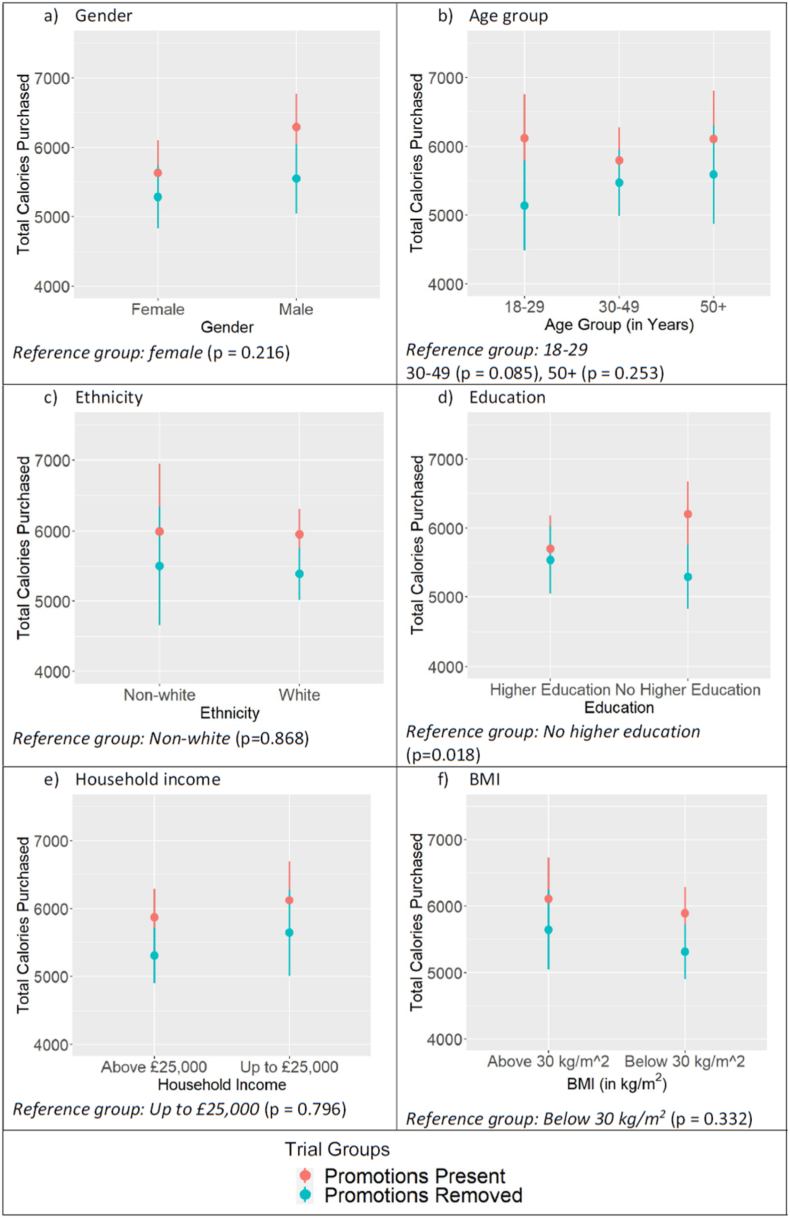
Estimated marginal means of impact on energy selected (kcal) by trial group and demographic. The stated p-value is for the interaction term between trial group and demographic. See Supplementary Tables S4, S14 – S18 for full regression models, and Supplementary Figs. S1–S6 for secondary outcome plots.

There was no evidence of a significant change in energy purchased when promotions were applied in any one specific food category, but there was reduced power for these analyses and the point estimate ranged from approximately 100 to 200 kcals lower in each category when promotions were removed (see Table 3). There were no significant interactions by demographic group at the pre-specified threshold of p < 0.003, but subgroup analyses were suggestive of the effect being stronger for people in the A-levels or lower education category (p = 0.018) (Fig. 2/Supplemental Table S18).

**Table 3:** Shop characteristics and comparison of energy selected (kcal) between trial groups by food category. See Supplementary Tables S10–S13 for full regression models.

		Confectionery	Crisps, Nuts, and Snacking Fruit	Biscuits and crackers	Cakes
Percent of items promoted in Promotions Present condition		31%	17%	18%	15%
Mean (SD) kcal per item purchased		548 (452)	730 (456)	663 (578)	592 (560)
Mean items purchased from category per participant (N = 511)		1.5 (1.8)	2.4 (1.6)	1.2 (1.2)	1.0 (1.0)
No of participants purchasing from category		358	445	341	315
Mean (SD) energy selected (kcal)	Total	1485 (923)	2199 (1394)	1776 (1070)	1574 (887)
	Promotions present	1581 (977)	2280 (1309)	1828 (1064)	1640 (912)
	Promotions removed	1385 (856)	2115 (1476)	1724 (1077)	1502 (856)
Comparison of energy selected (kcal)	Coefficient (95% CI)	−192 (−383, −1)	−186 (−425, 53)	−103 (−331, 126)	−137 (−333, 58)
	Coefficient p-value	0.049	0.159	0.378	0.170

*Reference group: promotions present.* *p < 0.003 – the threshold for significance following Bonferroni adjustment for secondary outcomes.

Following the shopping task, participants were asked about the most important factors driving their food choices. In both groups, price and taste (or preference) were most often cited as the top factors in selecting food for purchase (selected by 28–30% of participants; see Supplementary Table S5). The majority of participants also reported being more influenced by price reductions than multi-buy promotions (67% in promotions present group and 56% in promotions removed group; see Supplementary Table S7). When asked to consider whether or not they would support or oppose upcoming legislation to restrict HFSS multi-buys, about 12.5% of all participants neither supported nor opposed the legislation, 32% opposed it to some degree, and 55.5% supported it to some degree (see Supplementary Table S6).

## 4 Discussion

This randomised trial provided evidence that when promotions are removed on HFSS confectionery and snack foods, the total energy content of selected foods is 10% lower. There was no evidence of a statistically significant difference between trial groups in individual categories of food. There were no significant interactions with age, gender, ethnicity, income or education, with no evidence to suggest this policy would exacerbate health inequalities. There was no evidence of any differences in the relative proportion of particular nutrients in baskets.

This online experimental supermarket can test the effectiveness of potential changes to online grocery environments in a relatively naturalistic setting, with objective measures of behaviour. However, there are some limitations inherent in the design. Data for the setup of the experimental supermarket platform came from the most recent extract of foodDB available, which was April 2019. Therefore, it was not possible to know if the products participants selected would still be available or in stock at the time of the study. For this reason, participants in the study did not actually receive the foods they selected, but were reimbursed for their time instead. Participants may not make the same decisions they would if they were spending their own money or taking the products home to consume. However, it is likely that if participants were spending personal money then price promotions may be more salient. There is a risk that some participants may have bought more in the promotions removed condition just because items were cheaper, if they were shopping up to the maximum budget rather than following the task to choose snacks for friends realistically; however, the hypothesis that people may buy more just because it is cheaper formed an inherent part of what this study set out to test. Analysing spend suggested little evidence that participants were maximising their budgets (£10), with the mean cost of items in shopping baskets for the control group at £8.72, and £9.06 for the intervention. Study participants were instructed what type of foods they should purchase, with a focus on HFSS categories. The magnitude of the difference observed here may not reflect those that would occur in alternative shopping scenarios, such as a weekly shop and may not be consistent in physical shopping environments. Although there are limits on the external validity of this experimental design it can provide proof of concept evidence of the direction of effects and possible subgroup differences if similar policies were introduced. This is important since it is otherwise extremely difficult to test policies ahead of legislative changes.

This study complements previous literature on how promotions influence purchasing patterns and provides novel experimental evidence on the extent to which the removal of promotions on HFSS products may influence purchasing behaviours. Systematic reviews have previously suggested that reductions in food prices and/or the addition of promotions on healthy food stimulate purchases of healthier foods, such as fruits and vegetables especially when done in combination with other positioning or availability changes (Adam & Jensen, 2016; Bennett et al., 2020; Escaron et al., 2013; Gittelsohn et al., 2017; Watt et al., 2020). However, most previous experimental studies chose to promote healthy food and only a few looked at raising the prices or taxes on unhealthy products. This is in spite of evidence that promotions are more frequently applied on less healthy products, with recent data suggesting over 80% of products on promotional offer in advertising leaflets were unhealthy products, and in four of the leading UK supermarkets, approximately half of all promoted products were unhealthy products (Bennett et al., 2020; Gittelsohn et al., 2017; Superlist Health II provides insight, 2022; Watt et al., 2020; Haan, van Engen-Cocquyt, Finbow, Winkel, & Brandsma, 2021).

It has been suggested that price promotions could be a more equitable way of implementing a dietary intervention than other individual-targeted interventions, such as health education campaigns (Martin, Bauld, & Angus, 2017). Overall, we found no clear evidence of significant differences in the effectiveness of the intervention by participant characteristics or interaction effects between the groups and the intervention. However, the pattern of results for education was suggestive that removing promotions might be less impactful for those with a degree or higher, showing a greater effect in the reference group, those with secondary education or less as their highest education attained. Studies have shown that educational status is an indicator of diet quality inequalities (Rippin et al., 2020). This requires testing in a better-powered study, where a larger sample could also allow for more attribute levels for all demographic variables (i.e. groups for no education completed, GCSEs only, A-levels only, bachelor's only, etc.), but, if replicated, could suggest that targeting price promotions could help counteract diet-related inequalities.

In July 2021, the government in England passed legislation (Department of Health and Social Care, 2021a) yet to be implemented, restricting multi-buy promotions on unhealthy foods, such as HFSS confectionery and snacks (Department of Health and Social Care, 2021b). The findings of this study highlight the potential of the legislation to reduce purchasing of HFSS foods, as well as the potential favourability of the legislation, with 55.5% of study participants expressing some degree of support for the legislation. However, due to the experimental supermarket design capabilities, this study was focused on removal of temporary price reductions rather than multi-buy promotions targeted in the legislation. In practice it is very likely that retailers will change their promotional behaviour in response to legislation to restrict multi-buys. This was previously seen in response to the multi-buy ban on alcohol in Scotland, where there was a compensatory rise in temporary price reductions (Nakamura et al., 2014). Indeed, it has been reported that some grocery stores have already changed the balance of their promotions in favour of price reductions over multi-buy offers (Haan, van Engen-Cocquyt, Finbow, Winkel, & Brandsma, 2021). Research to evaluate these policies when enacted will be crucial to study the compensatory behaviours and to inform future legislative action.

## 5 Conclusion

Removing promotions on HFSS confectionery and snack foods resulted in less total energy being selected in an online experimental supermarket study. This provides evidence of the potential effectiveness of the forthcoming legislation in England to limit promotions on these food categories and reduce the risk of overconsumption.

## Funding

This research was funded by the NIHR Applied Research Collaboration (Oxford and Thames Valley). SAJ is also funded by the National Institute of Health Research Oxford Biomedical Research Centre (BRC) Obesity, Diet and Lifestyle Theme. RP is funded by a Royal Society and Wellcome Trust Sir Henry Dale fellowship (222566/Z/21/Z). CP is funded by the Ramon y Cajal Fellowship, Spanish State Plan for Scientific and Technical Research and Innovation 2017-2020 (RYC2020-028818-I). The funders had no role in the study design, data collection, analysis or interpretation. For the purpose of Open Access, the author has applied a CC BY public copyright licence to any Author Accepted Manuscript version arising from this submission.

## Data Availability

The dataset generated and analysed during the current study is available in the Open Science Framework repository: https://osf.io/9ru3e/.
