# Supplementary material for: The impact of price promotions on confectionery and snacks on the energy content of shopping baskets: A randomised controlled trial in an experimental online supermarket
Source: Appetite. 2023 Jul 1;186:106539. doi: 10.1016/j.appet.2023.106539 (PMC10933760; doi:10.1016/j.appet.2023.106539)

**The impact of price promotions on confectionery and snacks on the energy content of shopping baskets: A randomised controlled trial in an experimental online supermarket**

**Supplementary Materials**

Contents

[**Table S1**. Percentage of promotions applied to specific product categories 2](#_Toc99354321)

[**Table S2**. Full descriptive statistics of study population 3](#_Toc99354322)

[**Table S3**. Characteristics of purchases by demographic 5](#_Toc99354323)

[**Table S4.** Moderation of impact of trial group on energy selected (kcal) by demographic 6](#_Toc99354324)

[**Table S5.** Post-task survey on the most important factors affecting choice of purchase of foods or drinks by trial arm (n, %) 7](#_Toc99354325)

[**Table S6.** Post-survey task asking if participants would support or oppose new legislation 8](#_Toc99354326)

[**Table S7.** Post-survey task asking participants to identify what promotions they were most influenced by when making purchases 9](#_Toc99354327)

[**Table S8.** Primary and secondary regression models 10](#_Toc99354328)

[**Table S9**. Sensitivity analysis regression models 12](#_Toc99354329)

[**Table S10**. Primary and secondary regression models for food purchased from the confectionery category 13](#_Toc99354330)

[**Table S11**. Primary and secondary regression models for food purchased from the crisps, nuts, and snacking fruit category 14](#_Toc99354331)

[**Table S12**. Primary and secondary regression models for food purchased from the biscuits and crackers category 15](#_Toc99354332)

[**Table S13**. Primary and secondary regression models for food purchased from the cakes and tarts category 16](#_Toc99354333)

[**Table S14**. Primary and secondary outcome regression models by ethnicity group 17](#_Toc99354334)

[**Table S15**. Primary and secondary outcome regression models by BMI group 19](#_Toc99354335)

[**Table S16**. Primary and secondary outcome regression models by education levels 21](#_Toc99354336)

[**Table S17**. Primary and secondary outcome regression models by household income level 23](#_Toc99354337)

[**Table S18**. Primary and secondary outcome regression models by age groups 25](#_Toc99354338)

[**Figure S1**. Marginalised mean plots for secondary outcomes by gender 27](#_Toc99354339)

[**Figure S2**. Marginalised mean plots for secondary outcomes by BMI groups 28](#_Toc99354340)

[**Figure S3**. Marginalised mean plots for secondary outcomes by education level 29](#_Toc99354341)

[**Figure S4**. Marginalised mean plots for secondary outcomes by household income 30](#_Toc99354342)

[**Figure S5**. Marginalised mean plots for secondary outcomes by ethnicity group 31](#_Toc99354343)

[**Figure S6**. Marginalised mean plots for secondary outcomes by age group 32](#_Toc99354344)

[**Figure S7.** Example of the experimental supermarket with promotions. 33](#_Toc99354345)

[**Figure S8.** Example of the experimental supermarket without promotions. 33](#_Toc99354346)

## **Table S1**. Percentage of promotions applied to specific product categories

| **General Category** | **Dataset categories** | **% of products w/promotions applied** |
| --- | --- | --- |
| **Confectionery** | Block chocolate | 27 |
|  | Boxed chocolate | 56 |
|  | Chocolate pouches & bags | 9 |
|  | Multipack chocolate | 8 |
|  | Single bar chocolate | 62 |
|  | Sweets | 31 |
| **Biscuits & crackers** | Biscuits | 19 |
|  | Cereal bars & breakfast biscuits | 28 |
|  | Crackers, crispbreads & nibbles | 11 |
|  | Cookies | 19 |
| **Crisps, nuts and snacking fruit** | Multipack crisps | 22 |
|  | Nuts | 9 |
|  | Popcorn | 36 |
|  | Sharing crisps | 27 |
|  | Single pack crisps | 0 |
|  | Snacking fruit & seeds | 2 |
| **Cakes and tarts, AND Birthday & party cakes** | Mini rolls & slices | 22 |
|  | Pies & tarts | 11 |
|  | Sharing bites | 25 |
|  | Tea time cakes | 8 |
|  | Birthday & party cakes | 9 |
|  | Cakes & slices | 25 |

## **Table S2**. Full descriptive statistics of study population

|  |  | **Control**  **(n =257)** | **Intervention**  **(n = 254)** | **Total** |
| --- | --- | --- | --- | --- |
| **Age (years) mean + SD** | | 38.83 + 12.64 | 38.73 + 13.12 | 38.78 + 12.87 |
| **Gender** | |  |  |  |
|  | Female | 131 | 138 | 269 |
|  | Male | 124 | 116 | 240 |
|  | Other | 2 | 0 | 2 |
|  | Total | 257 | 254 | 511 |
| **BMI** | |  |  |  |
|  | <18.5 | 5 | 5 | 10 |
|  | 18.5 - 24.99 | 102 | 98 | 200 |
|  | 25 - 29.99 | 74 | 70 | 144 |
|  | 30 - 39.99 | 57 | 57 | 114 |
|  | >40 | 18 | 23 | 41 |
|  | Missing | 1 | 1 | 2 |
|  | Total | 257 | 254 | 511 |
| **Ethnicity** | |  |  |  |
|  | Asian | 19 | 18 | 37 |
|  | Black | 7 | 12 | 19 |
|  | Mixed | 3 | 6 | 9 |
|  | Other | 3 | 4 | 7 |
|  | Prefer not to say | 0 | 1 | 1 |
|  | White | 225 | 213 | 438 |
|  | Total | 257 | 254 | 511 |
| **Education** | |  |  |  |
|  | None | 2 | 1 | 3 |
|  | Up to 4 GCSE | 28 | 20 | 48 |
|  | 5+ GCSE/ 1 A-Level | 36 | 44 | 80 |
|  | 2+ A-level | 65 | 70 | 135 |
|  | Bachelor's | 76 | 80 | 156 |
|  | Post-Grad | 50 | 39 | 89 |
|  | Total | 257 | 254 | 511 |
| **Income** | |  |  |  |
|  | Below 15.5k | 39 | 31 | 70 |
|  | 15.5k - 25k | 52 | 41 | 93 |
|  | 25,001 - 39,999 | 62 | 68 | 130 |
|  | Above 40k | 104 | 114 | 218 |
|  | Total | 257 | 254 | 511 |
| **Online Grocery Shopping Frequency** | |  |  |  |
|  | Never or not in last year | 76 | 63 | 139 |
|  | 1-3 times in last year | 54 | 57 | 111 |
|  | 4-11 times in last year | 63 | 55 | 118 |
|  | 1-3 times per month | 34 | 28 | 62 |
|  | Once per week or more often | 30 | 51 | 81 |
|  | Total | 257 | 254 | 511 |
| **Number of people in household (median, IQR)** | | 3.0 (2.0 - 4.0) | 3.0 (2.0 - 4.0) | 3.0 (2.0 -4.0) |
| **Number of people in household (mean + SD)** | | 2.94 + 1.38 | 2.94 + 1.25 | 2.94 + 1.32 |
| **Weekly Grocery Spending (median, IQR)** | | 63.00 (50.00 - 100.00) | 80.00 (50.00 - 100.00) | 70.00 (50.00 -100.00) |
| **Weekly Grocery Spending (mean + SD)** | | 76.36 + 44.66 | 83.03 + 42.55 | 79.68 + 43.71 |

## **Table S3**. Characteristics of purchases by demographic

| Demographic groups | | N | Mean No. of Items | Mean Spend | Sat. Fat (% energy) | Sugar (% energy) | Salt  (g/  100g) | Energy Density (kcal/ 100g) | Mean Energy (kcal) |
| --- | --- | --- | --- | --- | --- | --- | --- | --- | --- |
| Gender | Male | 240 | 7.20 (2.86) | 8.97 (1.95) | 14.54 (4.72) | 21.81 (9.78) | 0.89 (0.39) | 471  (137) | 5933 (2046) |
|  | Female | 269 | 6.82 (1.84) | 8.82 (1.20) | 14.39 (4.19) | 23.31 (9.33) | 0.84 (0.32) | 457  (118) | 5451 (1577) |
|  | Other | 2 | 7.50 (0.71) | 9.35 (0.37) | 16.36 (0.20) | 22.93 (7.12) | 1.16 (0.41) | 515  (77) | 6754  (410) |
| Ethnicity | Non-white | 73 | 7.07 (2.24) | 9.01 (1.43) | 14.81 (4.72) | 24.51 (9.23) | 0.85 (0.38) | 489  (180) | 5689 (1704) |
|  | White | 438 | 6.99 (2.40) | 8.87 (1.62) | 14.41 (4.39) | 22.28 (9.58) | 0.89 (0.35) | 460  (115) | 5681 (1846) |
| Education | No higher | 266 | 7.03 (2.54) | 8.81 (1.81) | 14.45 (4.71) | 22.16 (9.52) | 0.89 (0.34) | 468  (117) | 5744 (1960) |
|  | Higher | 245 | 6.97 (2.19) | 8.99 (1.32) | 14.49 (4.13) | 23.08 (9.59) | 0.84 (0.37) | 460  (136) | 5616 (1667) |
| Income | Up to £25k | 163 | 7.26 (2.85) | 8.96 (1.75) | 14.45 (4.93) | 23.34 (9.84) | 0.83 (0.33) | 471  (154) | 5863 (1931) |
|  | Above £25k | 348 | 6.88 (2.11) | 8.86 (1.52) | 14.48 (4.19) | 22.26 (9.41) | 0.88 (0.37) | 461  (112) | 5598 (1769) |
| Age Groups | 18-29 | 142 | 7.04 (2.60) | 8.73 (1.39) | 15.13 (4.41) | 26.01 (9.37) | 0.77 (0.33) | 454  (138) | 5606 (1820) |
|  | 30-49 | 253 | 6.98 (2.17) | 8.85 (1.48) | 14.18 (4.43) | 22.20 (8.91) | 0.89 (0.33) | 465  (114) | 5643 (1761) |
|  | 50+ | 116 | 6.99 (2.53) | 9.20 (1.98) | 14.29 (4.44) | 19.31 (9.87) | 0.93 (0.42) | 474  (138) | 5863 (1967) |
| BMI | Below 30 kg/m^2^ | 354 | 6.92 (2.34) | 8.82 (1.45) | 14.44 (4.48) | 22.85 (9.81) | 0.86 (0.36) | 464  (132) | 5603 (1753) |
|  | Above 30 kg/m^2^ | 155 | 7.23 (2.44) | 9.08 (1.85) | 14.62 (4.28) | 22.18 (8.85) | 0.87 (0.36) | 464  (116) | 5877 (1968) |

## **Table S4.** Moderation of impact of trial group on energy selected (kcal) by demographic

|  | **Demographic Reference Group Mean (SD)** | **Demographic Test Group Mean (SD)** | | | **Interaction Term** **with Study condition (Promotions removed)**  (95% CIs) | | | **p-value** |
| --- | --- | --- | --- | --- | --- | --- | --- | --- |
| **Demographic Groups** | | |  |  | |  |  |  |
| Gender (Male)  (*Reference: Female*) | 5451 (1577) | 5933 (2045) | | | -395 (201, 1078) | | | 0.216 |
| Age Group 30-49  (*Reference:18-29*) | 5606 (1820) | 5643 (1761) | | | 653 (-89, 1395) | | | 0.085 |
| Age Group 50+  (*Reference:18-29*) | 5606 (1820) | 5863 (1967) | | | 464 (-422, 1350) | | | 0.253 |
| Ethnicity (White)  (*Reference: Non-white*) | 5689 (1704) | 5681 (1846) | | | -76 (-978, 826) | | | 0.868 |
| Education (Higher) (*Reference: No Higher Education*) | 5744 (1960) | 5616 (1667) | | | 756 (132, 1381) | | | 0.018 |
| Household Income (Above £25k)  *(Reference: Up to £25,000*) | 5863 (1931) | 5598 (1769) | | | -89 (-764, 586) | | | 0.796 |
| BMI (Above 30 kg/m^2^)  (*Reference: Below 30 kg/m^2^*) | 5587 (1734) | 5737 (1764) | | | 116 (-567, 798) | | | 0.332 |

**Reference group: promotions present*; p<0.003 – the threshold for significance following Bonferroni adjustment

## **Table S5.** Post-task survey on the most important factors affecting choice of purchase of foods or drinks by trial arm (n, %)

| **Factor for choice** | **Promotions Present**  **(n, %)** | | **Promotions Removed (n, %)** | |
| --- | --- | --- | --- | --- |
| Appearance | 30 | 4.01 | 37 | 4.93 |
| Convenience | 52 | 6.94 | 42 | 5.59 |
| Habits | 53 | 7.08 | 47 | 6.26 |
| Healthiness | 53 | 7.08 | 71 | 9.45 |
| Organic | 3 | 0.40 | 4 | 0.53 |
| Other | 18 | 2.40 | 11 | 1.46 |
| Price | 225 | 30.04 | 226 | 30.09 |
| Special diet | 5 | 0.67 | 6 | 0.80 |
| Special offers | 83 | 11.08 | 96 | 12.78 |
| Taste (preference) | 227 | 30.31 | 211 | 28.10 |

*Respondents could select up to three. Promotions present (n = 749) and promotions removed (n = 751). Percentage is percent of responses.*

## **Table S6.** Post-survey task asking if participants would support or oppose new legislation

| **Response** | **Promotions Present (n, %)** | | **Promotions Removed (n, %)** | |
| --- | --- | --- | --- | --- |
| Strongly oppose | 18 | 7.00 | 24 | 9.49 |
| Oppose | 28 | 10.89 | 21 | 8.30 |
| Somewhat oppose | 39 | 15.18 | 33 | 13.04 |
| Neither support nor oppose | 31 | 12.06 | 33 | 13.04 |
| Somewhat support | 64 | 24.90 | 59 | 23.32 |
| Support | 41 | 15.95 | 51 | 20.16 |
| Strongly support | 36 | 14.01 | 32 | 12.65 |

*Promotions present total responses, n = 257. Promotions removed total responses, n = 253.*

## **Table S7.** Post-survey task asking participants to identify what promotions they were most influenced by when making purchases

| **Response** | **Promotions Present**  **(n, %)** | | **Promotions Removed**  **(n, %)** | |
| --- | --- | --- | --- | --- |
| Multi-buy | 49 | 19.07 | 69 | 27.27 |
| No difference | 34 | 13.23 | 40 | 15.81 |
| Price reduction | 171 | 66.54 | 141 | 55.73 |
| Don't know | 3 | 1.17 | 3 | 1.19 |

*Promotions present total responses, n = 257. Promotions removed total responses, n = 253.*

## **Table S8.** Primary and secondary regression models

|  | Coefficient | 95% CI | p-value | |
| --- | --- | --- | --- | --- |
| *Total Calories* |  |  |  | |
| **Cohort** |  |  |  | |
| Promotions Present | Reference |  |  | |
| Promotions Removed | -552 | (-866, -238) | 0.0006 | |
| **Weekly Grocery Spending** | 2.4 | (-1.24, 5.95) | 0.200 | |
| **Constant** | 5769 | (5417, 6122) | <0.0001 | |
| *Percent Energy from Saturated Fat* | | | |  |
| **Cohort** |  |  |  | |
| Promotions Present | Reference |  |  | |
| Promotions Removed | 0.17 | (-0.60, 0.94) | 0.671 | |
| **Weekly Grocery Spending** | -0.006 | (-0.014, 0.003) | 0.222 | |
| **Constant** | 14.82 | (13.96, 15.69) | <0.0001 | |
| *Calories from Saturated Fat* | | | |  |
| **Cohort** |  |  |  | |
| Promotions Present | Reference |  |  | |
| Promotions Removed | -71.3 | (-132, -11.0) | 0.021 | |
| **Weekly Grocery Spending** | 0.081 | (-0.61, 0.77) | 0.818 | |
| **Constant** | 846 | (779, 914) | <0.0001 | |
| *Grams from Saturated Fat* | | | |  |
| **Cohort** |  |  |  | |
| Promotions Present | Reference |  |  | |
| Promotions Removed | -7.92 | (-14.6, -1.22) | 0.021 | |
| **Weekly Grocery Spending** | 0.009 | (-0.068, 0.086) | 0.818 | |
| **Constant** | 94.0 | (86.5, 102) | <0.0001 | |
| *Percent energy from sugar* | | | |  |
| **Cohort** |  |  |  | |
| Promotions Present | Reference |  |  | |
| Promotions Removed | -0.48 | (-2.13, 1.18) | 0.574 | |
| **Weekly Grocery Spending** | -0.019 | (-0.038, 0.0004) | 0.055 | |
| **Constant** | 24.32 | (22.46, 26.18) | <0.0001 | |
| *Calories from Sugar* |  |  |  | |
| **Cohort** |  |  |  | |
| Promotions Present | Reference |  |  | |
| Promotions Removed | -158.6 | (-274, -43.4) | 0.007 | |
| **Weekly Grocery Spending** | -0.91 | (-2.23, 0.40) | 0.175 | |
| **Constant** | 1440 | (1311, 1570) | <0.0001 | |
| *Grams from Sugar* |  |  |  | |
| **Cohort** |  |  |  | |
| Promotions Present | Reference |  |  | |
| Promotions Removed | -39.64 | (-68.4, -10.9) | 0.007 | |
| **Weekly Grocery Spending** | -0.23 | (-0.56, 0.10) | 0.175 | |
| **Constant** | 360 | (328, 392) | <0.0001 | |
| *Kcal/100g* |  |  |  | |
| **Cohort** |  |  |  | |
| Promotions Present | Reference |  |  | |
| Promotions Removed | -13.2 | (-35.3, 8.81) | 0.240 | |
| **Weekly Grocery Spending** | 0.049 | (-0.20, 0.30) | 0.704 | |
| **Constant** | 467 | (442, 492) | <0.0001 | |

## **Table S9**. Sensitivity analysis regression models

|  | Coefficient | 95% CI | p-value | |
| --- | --- | --- | --- | --- |
| *Total Calories* |  |  |  | |
| **Cohort** |  |  |  | |
| Promotions Present | Reference |  |  | |
| Promotions Removed | -693 | (-1014, -373) | <0.0001 | |
| **Weekly Grocery Spending** | 0.82 | (-2.9, 4.5) | 0.439 | |
| **Constant** | 6139 | (5779, 6500) | <0.0001 | |
| *Percent Energy from Saturated Fat* | | | |  |
| **Cohort** |  |  |  | |
| Promotions Present | Reference |  |  | |
| Promotions Removed | 0.39 | (-0.42, 1.21) | 0.342 | |
| **Weekly Grocery Spending** | -0.0046 | (-0.014, 0.005) | 0.331 | |
| **Constant** | 14.74 | (13.82, 15.65) | <0.0001 | |
| *Percent energy from sugar* | | | |  |
| **Cohort** |  |  |  | |
| Promotions Present | Reference |  |  | |
| Promotions Removed | -0.54 | (-2.28, 1.19) | 0.541 | |
| **Weekly Grocery Spending** | -0.019 | (-0.038, 0.001) | 0.065 | |
| **Constant** | 24.46 | (23.51, 27.42) | <0.0001 | |
| *Salt g/100g* |  |  |  | |
| **Cohort** |  |  |  | |
| Promotions Present | Reference |  |  | |
| Promotions Removed | -0.043 | (-0.11, 0.02) | 0.179 | |
| Weekly Grocery Spending | 0.001 | (0.0004, 0.002) | 0.002 | |
| Constant | 0.79 | (-0.11, 0.020) | <0.0001 | |
| *Kcal/100g* |  |  |  | |
| **Cohort** |  |  |  | |
| Promotions Present | Reference |  |  | |
| Promotions Removed | -5.87 | (-26.6, 14.9) | 0.580 | |
| **Weekly Grocery Spending** | 0.029 | (-0.21, 0.27) | 0.809 | |
| **Constant** | 492 | (468, 515) | <0.0001 | |

## **Table S10**. Primary and secondary regression models for food purchased from the confectionery category

|  | Coefficient | 95% CI | p-value | |
| --- | --- | --- | --- | --- |
| *Total Calories* |  |  |  | |
| **Cohort** |  |  |  | |
| Promotions Present | Reference |  |  | |
| Promotions Removed | -192 | (-383, -1) | 0.050 | |
| **Weekly Grocery Spending** | -0.65 | (-2.79, 1.49) | 0.553 | |
| **Constant** | 1631 | (1420, 1842) | <0.0001 | |
| *Percent Energy from Saturated Fat* | | | |  |
| **Cohort** |  |  |  | |
| Promotions Present | Reference |  |  | |
| Promotions Removed | 0.50 | (-0.50, 1.50) | 0.329 | |
| **Weekly Grocery Spending** | -0.003 | (-0.01, 0.008) | 0.548 | |
| **Constant** | 5.98 | (4.90, 7.07) | <0.0001 | |
| *Percent energy from sugar* | | | |  |
| **Cohort** |  |  |  | |
| Promotions Present | Reference |  |  | |
| Promotions Removed | -0.007 | (-1.8, 1.8) | 0.993 | |
| **Weekly Grocery Spending** | -0.007 | (-0.03, 0.01) | 0.465 | |
| **Constant** | 14.0 | (11.99, 15.95) | <0.0001 | |
| *Salt g/100g* |  |  |  | |
| **Cohort** |  |  |  | |
| Promotions Present | Reference |  |  | |
| Promotions Removed | -0.0009 | (-0.013, 0.011) | 0.883 | |
| Weekly Grocery Spending | -0.000059 | (-0.0002, 0.000071) | 0.369 | |
| Constant | 0.075 | (0.063, 0.088) | <0.0001 | |
| *Kcal/100g* |  |  |  | |
| **Cohort** |  |  |  | |
| Promotions Present | Reference |  |  | |
| Promotions Removed | -0.096 | (-17.74, 17.55) | 0.992 | |
| **Weekly Grocery Spending** | -0.067 | (-0.26, 0.13) | 0.508 | |
| **Constant** | 136 | (116, 155) | <0.0001 | |

## **Table S11**. Primary and secondary regression models for food purchased from the crisps, nuts, and snacking fruit category

|  | Coefficient | 95% CI | p-value |
| --- | --- | --- | --- |
| *Total Calories* |  |  |  |
| **Cohort** |  |  |  |
| Promotions Present | Reference |  |  |
| Promotions Removed | -186 | (-425, 53) | 0.159 |
| **Weekly Grocery Spending** | 3.2 | (0.33, 6.13) | 0.030 |
| **Constant** | 2027 | (1737, 2317) | <0.0001 |
| *Percent Energy from Saturated Fat* | | | |
| **Cohort** |  |  |  |
| Promotions Present | Reference |  |  |
| Promotions Removed | 0.069 | (-0.41, 0.28) | 0.329 |
| **Weekly Grocery Spending** | 0.003 | (-0.001, 0.007) | 0.548 |
| **Constant** | 2.45 | (2.06, 2.83) | <0.0001 |
| *Percent energy from sugar* | | | |
| **Cohort** |  |  |  |
| Promotions Present | Reference |  |  |
| Promotions Removed | 0.035 | (-0.62, 0.69) | 0.918 |
| **Weekly Grocery Spending** | -0.001 | (-0.009, 0.006) | 0.741 |
| **Constant** | 2.47 | (1.73, 3.21) | <0.0001 |
| *Salt g/100g* |  |  |  |
| **Cohort** |  |  |  |
| Promotions Present | Reference |  |  |
| Promotions Removed | -0.05 | (-0.11, 0.01) | 0.115 |
| Weekly Grocery Spending | 0.0007 | (-0.000025, 0.001) | 0.059 |
| Constant | 0.494 | (0.424, 0.564) | <0.0001 |
| *Kcal/100g* |  |  |  |
| **Cohort** |  |  |  |
| Promotions Present | Reference |  |  |
| Promotions Removed | -3.9 | (-25.72, 17.91) | 0.726 |
| **Weekly Grocery Spending** | 0.17 | (-0.072, 0.42) | 0.167 |
| **Constant** | 172 | (147, 196) | <0.0001 |

## **Table S12**. Primary and secondary regression models for food purchased from the biscuits and crackers category

|  | Coefficient | 95% CI | p-value | |
| --- | --- | --- | --- | --- |
| *Total Calories* |  |  |  | |
| **Cohort** |  |  |  | |
| Promotions Present | Reference |  |  | |
| Promotions Removed | -103 | (-331, 126) | 0.378 | |
| **Weekly Grocery Spending** | -0.23 | (-2.75, 2.29) | 0.857 | |
| **Constant** | 1846 | (1596, 2096) | <0.0001 | |
| *Percent Energy from Saturated Fat* | | | |  |
| **Cohort** |  |  |  | |
| Promotions Present | Reference |  |  | |
| Promotions Removed | 0.43 | (-0.40, 1.27) | 0.313 | |
| **Weekly Grocery Spending** | -0.003 | (-0.013, 0.006) | 0.471 | |
| **Constant** | 5.80 | (4.89, 6.72) | <0.0001 | |
| *Percent energy from sugar* | | | |  |
| **Cohort** |  |  |  | |
| Promotions Present | Reference |  |  | |
| Promotions Removed | 0.029 | (-1.21, 1.27) | 0.963 | |
| **Weekly Grocery Spending** | -0.012 | (-0.025, 0.002) | 0.098 | |
| **Constant** | 7.74 | (6.39, 9.10) | <0.0001 | |
| *Salt g/100g* |  |  |  | |
| **Cohort** |  |  |  | |
| Promotions Present | Reference |  |  | |
| Promotions Removed | 0.013 | (-0.024, 0.050) | 0.490 | |
| Weekly Grocery Spending | 0.0003 | (-0.0002, 0.0007) | 0.232 | |
| Constant | 0.22 | (0.18, 0.26) | <0.0001 | |
| *Kcal/100g* |  |  |  | |
| **Cohort** |  |  |  | |
| Promotions Present | Reference |  |  | |
| Promotions Removed | 4.4 | (-13.48, 22.29) | 0.629 | |
| **Weekly Grocery Spending** | -0.01 | (-0.21, 0.19) | 0.925 | |
| **Constant** | 140 | (121, 160) | <0.0001 | |

## **Table S13**. Primary and secondary regression models for food purchased from the cakes and tarts category

|  | Coefficient | 95% CI | p-value |
| --- | --- | --- | --- |
| *Total Calories* |  |  |  |
| **Cohort** |  |  |  |
| Promotions Present | Reference |  |  |
| Promotions Removed | -137 | (-333, 58) | 0.170 |
| **Weekly Grocery Spending** | -1.8 | (-4.2, 0.61) | 0.144 |
| **Constant** | 1781 | (1549, 2013) | <0.0001 |
| *Percent Energy from Saturated Fat* | | | |
| **Cohort** |  |  |  |
| Promotions Present | Reference |  |  |
| Promotions Removed | 0.017 | (-0.69, 0.73) | 0.963 |
| **Weekly Grocery Spending** | -0.004 | (-0.012, 0.005) | 0.429 |
| **Constant** | 5.36 | (4.52, 6.20) | <0.0001 |
| *Percent energy from sugar* | | | |
| **Cohort** |  |  |  |
| Promotions Present | Reference |  |  |
| Promotions Removed | -0.085 | (-1.36, 1.19) | 0.896 |
| **Weekly Grocery Spending** | -0.017 | (-0.033, -0.001) | 0.037 |
| **Constant** | 11.03 | (9.52, 12.53) | <0.0001 |
| *Salt g/100g* |  |  |  |
| **Cohort** |  |  |  |
| Promotions Present | Reference |  |  |
| Promotions Removed | 0.008 | (-0.009, 0.026) | 0.362 |
| Weekly Grocery Spending | -0.0002 | (-0.0004, 5.8*10^-5^) | 0.151 |
| Constant | 0.127 | (0.106, 0.148) | <0.0001 |
| *Kcal/100g* |  |  |  |
| **Cohort** |  |  |  |
| Promotions Present | Reference |  |  |
| Promotions Removed | -4.8 | (-22.10,12.43) | 0.584 |
| **Weekly Grocery Spending** | -0.20 | (-0.41, 0.012) | 0.065 |
| **Constant** | 144 | (124, 165) | <0.0001 |

## **Table S14**. Primary and secondary outcome regression models by ethnicity group

|  | Coefficient | 95% CI | p-value | |
| --- | --- | --- | --- | --- |
| *Total Calories* |  |  |  | |
| **Cohort** |  |  |  | |
| Promotions Present | Reference |  |  | |
| Promotions Removed | -489 | (-1327, 348) | 0.252 | |
| **Ethnicity** |  |  |  | |
| Non-white | Reference |  |  | |
| White | -38 | (-710, 634) | 0.912 | |
| **Promotions Removed : White** | -76 | (-979, 826) | 0.868 | |
| **Weekly Grocery Spending** | 2.4 | (-1.2, 6.0) | 0.192 | |
| **Constant** | 5798 | (5131, 6465) | <0.0001 | |
| *Percent Energy from Saturated Fat* | | | |  |
| **Cohort** |  |  |  | |
| Promotions Present | Reference |  |  | |
| Promotions Removed | -0.24 | (-2.30, 1.82) | 0.818 | |
| **Ethnicity** |  |  |  | |
| Non-white | Reference |  |  | |
| White | -0.57 | (-2.22, 1.08) | 0.497 | |
| **Promotions Removed : White** | 0.46 | (-1.76, 2.68) | 0.685 | |
| **Weekly Grocery Spending** | -0.005 | (-0.014, 0.004) | 0.252 | |
| **Constant** | 15.30 | (13.66, 16.94) | <0.0001 | |
| *Percent energy from sugar* | | | |  |
| **Cohort** |  |  |  | |
| Promotions Present | Reference |  |  | |
| Promotions Removed | -0.72 | (-5.13, 3.68) | 0.748 | |
| **Ethnicity** |  |  |  | |
| Non-white | Reference |  |  | |
| White | -2.16 | (-5.70,1.38) | 0.232 | |
| **Promotions Removed : White** | 0.19 | (-4.56, 4.94) | 0.939 | |
| **Weekly Grocery Spending** | -0.017 | (-0.036, 0.002) | 0.084 | |
| **Constant** | 26.08 | (22.57, 29.59) | <0.0001 | |
| *Salt g/100g* |  |  |  | |
| **Cohort** |  |  |  | |
| Promotions Present | Reference |  |  | |
| Promotions Removed | -0.19 | (-0.35, -0.023) | 0.026 | |
| **Ethnicity** |  |  |  | |
| Non-white | Reference |  |  | |
| White | -0.078 | (-0.21, 0.053) | 0.244 | |
| **Promotions Removed : White** | 0.15 | (-0.027, 0.33) | 0.097 | |
| **Weekly Grocery Spending** | 0.0011 | (0.0003, 0.0018) | 0.003 | |
| **Constant** | 0.87 | (0.74, 1.01) | <0.0001 | |
| *Kcal/100g* |  |  |  | |
| **Cohort** |  |  |  | |
| Promotions Present | Reference |  |  | |
| Promotions Removed | -70.15 | (-128.5, -11.8) | 0.019 | |
| **Ethnicity** |  |  |  | |
| Non-white | Reference |  |  | |
| White | -66.59 | (-113.5, -19.7) | 0.006 | |
| **Promotions Removed : White** | 64.68 | (1.77, 127.6) | 0.044 | |
| **Weekly Grocery Spending** | 0.079 | (-0.17, 0.33) | 0.538 | |
| **Constant** | 523 | (476, 569) | <0.0001 | |

## **Table S15**. Primary and secondary outcome regression models by BMI group

|  | Coefficient | 95% CI | p-value |
| --- | --- | --- | --- |
| *Total Calories* |  |  |  |
| **Cohort** |  |  |  |
| Promotions Present | Reference |  |  |
| Promotions Removed | -550 | (-912, -187) | 0.003 |
| **BMI** |  |  |  |
| Below 30 kg/m^2^ | Reference |  |  |
| Above 30 kg/m^2^ | 33.6 | (-463, 530) | 0.895 |
| **Promotions Removed : Above 30 kg/m^2^** | 249 | (-449, 947) | 0.485 |
| **Weekly Grocery Spending** | 2.08 | (-1.53, 5.68) | 0.259 |
| **Constant** | 5694 | (5325, 6063) | <0.0001 |
| *Percent Energy from Saturated Fat* | | | |
| **Cohort** |  |  |  |
| Promotions Present | Reference |  |  |
| Promotions Removed | -0.12 | (-1.03, 0.80) | 0.805 |
| **BMI** |  |  |  |
| Below 30 kg/m^2^ | Reference |  |  |
| Above 30 kg/m^2^ | 0.23 | (-1.02, 1.48) | 0.716 |
| **Promotions Removed : Above 30 kg/m^2^** | 0.33 | (-1.43, 2.09) | 0.710 |
| **Weekly Grocery Spending** | -0.005 | (-0.014, 0.004) | 0.247 |
| **Constant** | 14.95 | (14.02, 15.88) | <0.0001 |
| *Percent energy from sugar* | | | |
| **Cohort** |  |  |  |
| Promotions Present | Reference |  |  |
| Promotions Removed | 0.11 | (-1.88, 2.10) | 0.914 |
| **BMI** |  |  |  |
| Below 30 kg/m^2^ | Reference |  |  |
| Above 30 kg/m^2^ | 0.38 | (-2.35, 3.11) | 0.785 |
| **Promotions Removed : Above 30 kg/m^2^** | -2.45 | (-6.29, 1.38) | 0.210 |
| **Weekly Grocery Spending** | -0.017 | (-0.04, 0.003) | 0.090 |
| **Constant** | 24.18 | (22.15, 26.20) | <0.0001 |
| *Salt g/100g* |  |  |  |
| **Cohort** |  |  |  |
| Promotions Present | Reference |  |  |
| Promotions Removed | -0.070 | (-0.14, 0.001) | 0.055 |
| **BMI** |  |  |  |
| Below 30 kg/m^2^ | Reference |  |  |
| Above 30 kg/m^2^ | -0.020 | (-0.12, 0.078) | 0.684 |
| **Promotions Removed : Above 30 kg/m^2^** | 0.048 | (-0.090, 0.19) | 0.496 |
| **Weekly Grocery Spending** | 0.001 | (0.0004, 0.002) | 0.003 |
| **Constant** | 0.81 | (0.74, 0.88) | <0.0001 |
| *Kcal/100g* |  |  |  |
| **Cohort** |  |  |  |
| Promotions Present | Reference |  |  |
| Promotions Removed | -13.02 | (-39.26, 13.23) | 0.331 |
| **BMI** |  |  |  |
| Below 30 kg/m^2^ | Reference |  |  |
| Above 30 kg/m^2^ | -3.71 | (-39.65, 32.23) | 0.840 |
| **Promotions Removed : Above 30 kg/m^2^** | -7.59 | (-58.10, 42.92) | 0.768 |
| **Weekly Grocery Spending** | 0.011 | (-0.25, 0.27) | 0.934 |
| **Constant** | 470 | (443, 496) | <0.0001 |

## **Table S16**. Primary and secondary outcome regression models by education levels

|  | Coefficient | 95% CI | p-value |
| --- | --- | --- | --- |
| *Total Calories* |  |  |  |
| **Cohort** |  |  |  |
| Promotions Present | Reference |  |  |
| Promotions Removed | -917 | (-1351, 484) | <0.0001 |
| **Education** |  |  |  |
| No higher education | Reference |  |  |
| Higher education | -505 | (-945, -65.7) | 0.025 |
| **Promotions Removed : Higher education** | 756 | (132, 1381) | 0.018 |
| **Weekly Grocery Spending** | 2.4 | (-1.16, 6.01) | 0.186 |
| **Constant** | 6012 | (5598, 6426) | <0.0001 |
| *Percent Energy from Saturated Fat* | | | |
| **Cohort** |  |  |  |
| Promotions Present | Reference |  |  |
| Promotions Removed | 0.43 | (-0.64, 1.50) | 0.435 |
| **Education** |  |  |  |
| No higher education | Reference |  |  |
| Higher education | 0.29 | (-0.80, 1.38) | 0.600 |
| **Promotions Removed : Higher education** | -0.54 | (-2.08, 1.00) | 0.494 |
| **Weekly Grocery Spending** | -0.006 | (-0.014, 0.003) | 0.217 |
| **Constant** | 14.69 | (13.66, 15.71) | <0.0001 |
| *Percent energy from sugar* | | | |
| **Cohort** |  |  |  |
| Promotions Present | Reference |  |  |
| Promotions Removed | -0.050 | (-0.12, 0.054) | 0.482 |
| **Education** |  |  |  |
| No higher education | Reference |  |  |
| Higher education | -0.010 | (-0.096, 0.077) | 0.823 |
| **Promotions Removed : Higher education** | -0.059 | (-0.18, 0.064) | 0.349 |
| **Weekly Grocery Spending** | 0.0011 | (0.0004, 0.002) | 0.003 |
| **Constant** | 0.81 | (0.73, 0.90) | <0.0001 |
| *Salt g/100g* |  |  |  |
| **Cohort** |  |  |  |
| Promotions Present | Reference |  |  |
| Promotions Removed | -0.031 | (-0.12, 0.055) | 0.482 |
| **Education** |  |  |  |
| No higher education | Reference |  |  |
| Higher education | -0.010 | (-0.096, 0.077) | 0.823 |
| **Promotions Removed : Higher education** | -0.059 | (-0.18, 0.064) | 0.349 |
| **Weekly Grocery Spending** | 0.001 | (0.0004, 0.002) | 0.003 |
| **Constant** | 0.81 | (0.73, 0.90) | <0.0001 |
| *Kcal/100g* |  |  |  |
| **Cohort** |  |  |  |
| Promotions Present | Reference |  |  |
| Promotions Removed | -5.16 | (-35.76, 25.44) | 0.741 |
| **Education** |  |  |  |
| No higher education | Reference |  |  |
| Higher education | -0.29 | (-31.33, 30.76) | 0.986 |
| **Promotions Removed : Higher education** | -17.17 | (-61.25, 26.93) | 0.446 |
| **Weekly Grocery Spending** | 0.041 | (-0.21, 0.29) | 0.751 |
| **Constant** | 498 | (438, 497) | <0.0001 |

## **Table S17**. Primary and secondary outcome regression models by household income level

|  | Coefficient | 95% CI | p-value |
| --- | --- | --- | --- |
| *Total Calories* |  |  |  |
| **Cohort** |  |  |  |
| Promotions Present | Reference |  |  |
| Promotions Removed | -475 | (-1034, 83.9) | 0.096 |
| **Household Income** |  |  |  |
| Up to £25,000 | Reference |  |  |
| Above £25,000 | -249 | (-719, 222) | 0.300 |
| **Promotions Removed : Above £25,000** | -89.2 | (-764, 586) | 0.796 |
| **Weekly Grocery Spending** | 3.06 | (-0.63, 6.76) | 0.105 |
| **Constant** | 5876 | (5443, 6309) | <0.0001 |
| *Percent Energy from Saturated Fat* | | | |
| **Cohort** |  |  |  |
| Promotions Present | Reference |  |  |
| Promotions Removed | -0.73 | (-2.10, 0.64) | 0.299 |
| **Household Income** |  |  |  |
| Up to £25,000 | Reference |  |  |
| Above £25,000 | -0.47 | (-1.62, 0.69) | 0.429 |
| **Promotions Removed : Above £25,000** | 1.30 | (-0.36, 2.96) | 0.126 |
| **Weekly Grocery Spending** | -0.006 | (-0.015, 0.003) | 0.224 |
| **Constant** | 15.13 | (14.07, 16.20) | <0.0001 |
| *Percent energy from sugar* | | | |
| **Cohort** |  |  |  |
| Promotions Present | Reference |  |  |
| Promotions Removed | -3.28 | (-6.22, -0.34) | 0.029 |
| **Household Income** |  |  |  |
| Up to £25,000 | Reference |  |  |
| Above £25,000 | -2.62 | (-5.10, -0.15) | 0.038 |
| **Promotions Removed : Above £25,000** | 4.15 | (0.59, 7.70) | 0.023 |
| **Weekly Grocery Spending** | -0.016 | (-0.036, 0.003) | 0.103 |
| **Constant** | 25.83 | (23.55, 28.11) | <0.0001 |
| *Salt g/100g* |  |  |  |
| **Cohort** |  |  |  |
| Promotions Present | Reference |  |  |
| Promotions Removed | 0.0070 | (-0.10, 0.12) | 0.900 |
| **Household Income** |  |  |  |
| Up to £25,000 | Reference |  |  |
| Above £25,000 | 0.087 | (-0.0053, 0.18) | 0.065 |
| **Promotions Removed : Above £25,000** | -0.098 | (-0.23, 0.034) | 0.145 |
| **Weekly Grocery Spending** | 0.0010 | (0.0017, 0.0087) | 0.009 |
| **Constant** | 0.76 | (0.68, 0.84) | <0.0001 |
| *Kcal/100g* |  |  |  |
| **Cohort** |  |  |  |
| Promotions Present | Reference |  |  |
| Promotions Removed | -24.34 | (-63.65, 14.96) | 0.225 |
| **Household Income** |  |  |  |
| Up to £25,000 | Reference |  |  |
| Above £25,000 | -18.66 | (-51.73, 14.41) | 0.269 |
| **Promotions Removed : Above £25,000** | 17.05 | (-30.43, 64.53) | 0.482 |
| **Weekly Grocery Spending** | 0.079 | (-0.18, 0.34) | 0.554 |
| **Constant** | 477 | (446, 507) | <0.0001 |

## **Table S18**. Primary and secondary outcome regression models by age groups

|  | Coefficient | 95% CI | p-value |
| --- | --- | --- | --- |
| *Total Calories* |  |  |  |
| **Cohort** |  |  |  |
| Promotions Present | Reference |  |  |
| Promotions Removed | -978 | (-1572, -383) | 0.001 |
| **Age Group** |  |  |  |
| 18-29 y.o | Reference |  |  |
| 30-49 y.o | -322 | (-849, 204) | 0.231 |
| 50+ y.o. | -12.0 | (-638, 614) | 0.970 |
| **Promotions Removed : 30-49** | 653 | (-89.3, 1395) | 0.085 |
| **Promotions Removed: 50+** | 464 | (-422, 1350) | 0.305 |
| **Weekly Grocery Spending** | 2.16 | (-1.54, 5.86) | 0.253 |
| **Constant** | 5944 | (5472, 6415) | <0.0001 |
| *Percent Energy from Saturated Fat* | | | |
| **Cohort** |  |  |  |
| Promotions Present | Reference |  |  |
| Promotions Removed | 1.11 | (-0.35, 2.57) | 0.136 |
| **Age Group** |  |  |  |
| 18-29 y.o | Reference |  |  |
| 30-49 y.o | -0.015 | (-1.30, 1.28) | 0.982 |
| 50+ y.o. | -0.63 | (-2.16, 0.90) | 0.421 |
| **Promotions Removed : 30-49** | -1.77 | (-3.59, 0.051) | 0.057 |
| **Promotions Removed: 50+** | -0.31 | (-2.48, 1.86) | 0.777 |
| **Weekly Grocery Spending** | -0.0031 | (-0.012, 0.006) | 0.497 |
| **Constant** | 14.79 | (13.64, 15.95) | <0.0001 |
| *Percent energy from sugar* | | | |
| **Cohort** |  |  |  |
| Promotions Present | Reference |  |  |
| Promotions Removed | 0.22 | (-2.83, 3.27) | 0.888 |
| **Age Group** |  |  |  |
| 18-29 y.o | Reference |  |  |
| 30-49 y.o | -3.47 | (-6.18, -0.76) | 0.012 |
| 50+ y.o. | -5.23 | (-8.45, -2.02) | 0.002 |
| **Promotions Removed : 30-49** | -0.29 | (-4.10, 3.53) | 0.882 |
| **Promotions Removed: 50+** | -2.59 | (-7.15, 1.96) | 0.265 |
| **Weekly Grocery Spending** | -0.009 | (-0.028, 0.010) | 0.371 |
| **Constant** | 26.45 | (24.03, 28.87) | <0.0001 |
| *Salt g/100g* |  |  |  |
| **Cohort** |  |  |  |
| Promotions Present | Reference |  |  |
| Promotions Removed | -0.070 | (-0.19, -0.046) | 0.238 |
| **Age Group** |  |  |  |
| 18-29 y.o | Reference |  |  |
| 30-49 y.o | 0.096 | (-0.007, 0.20) | 0.067 |
| 50+ y.o. | 0.11 | (-0.013, 0.23) | 0.082 |
| **Promotions Removed : 30-49** | 0.0011 | (-0.14, 0.15) | 0.988 |
| **Promotions Removed: 50+** | 0.052 | (-0.12, 0.22) | 0.558 |
| **Weekly Grocery Spending** | 0.0008 | (0.0001, 0.002) | 0.021 |
| **Constant** | 0.75 | (0.66, 0.85) | <0.0001 |
| *Kcal/100g* |  |  |  |
| **Cohort** |  |  |  |
| Promotions Present | Reference |  |  |
| Promotions Removed | -48.87 | (-90.57, -7.17) | 0.022 |
| **Age Group** |  |  |  |
| 18-29 y.o | Reference |  |  |
| 30-49 y.o | -13.09 | (-50.04, 23.86) | 0.488 |
| 50+ y.o. | -6.05 | (-49.95, 37.86) | 0.787 |
| **Promotions Removed : 30-49** | 48.01 | (-4.07, 100.08) | 0.071 |
| **Promotions Removed: 50+** | 52.72 | (-9.44, 114.89) | 0.097 |
| **Weekly Grocery Spending** | 0.018 | (-0.24, 0.28) | 0.892 |
| **Constant** | 477 | (444, 510) | <0.0001 |

## **Figure S1**. Marginalised mean plots for secondary outcomes by gender

| 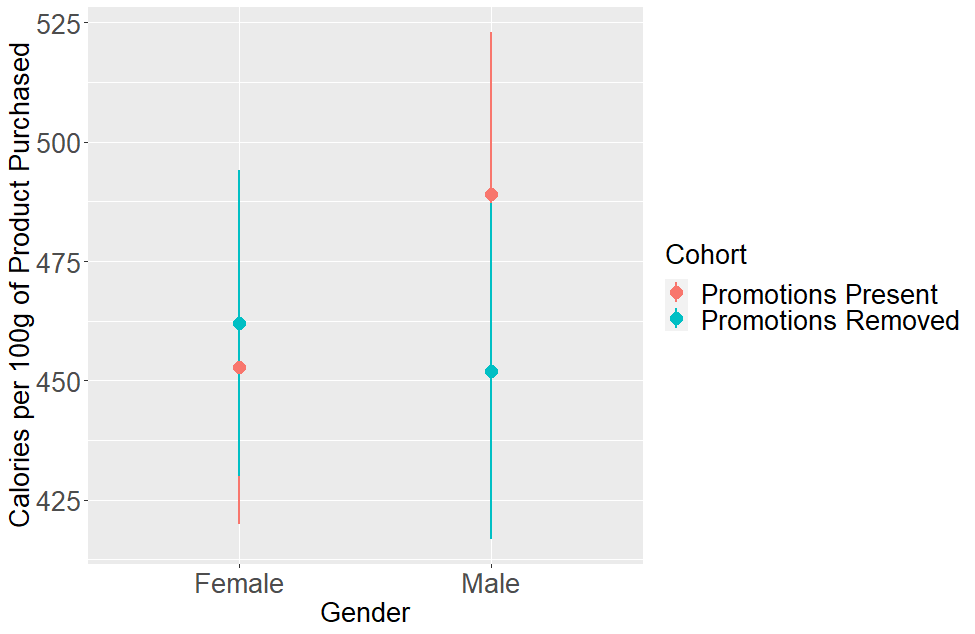 | 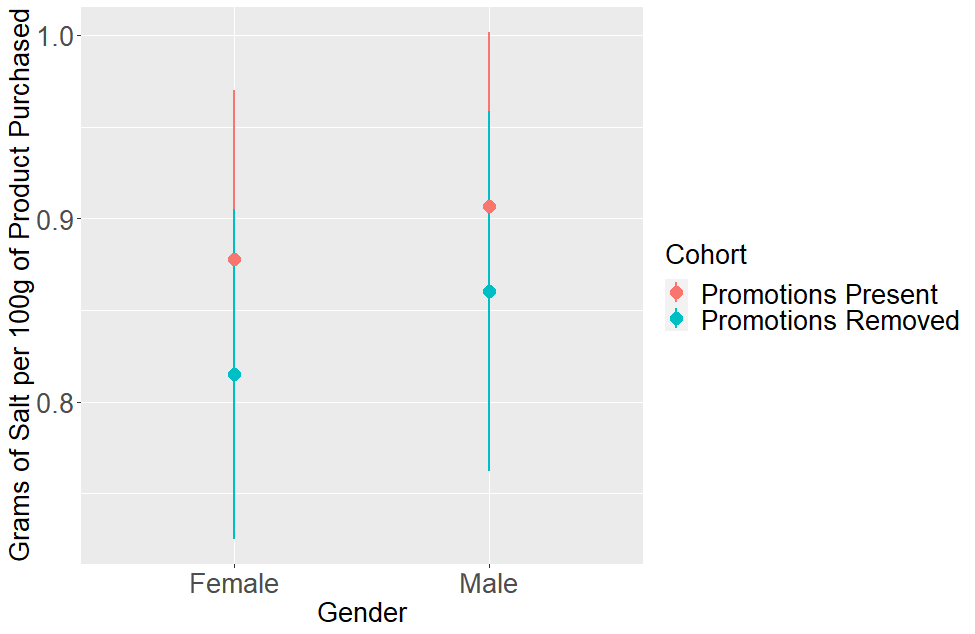 |
| --- | --- |
| 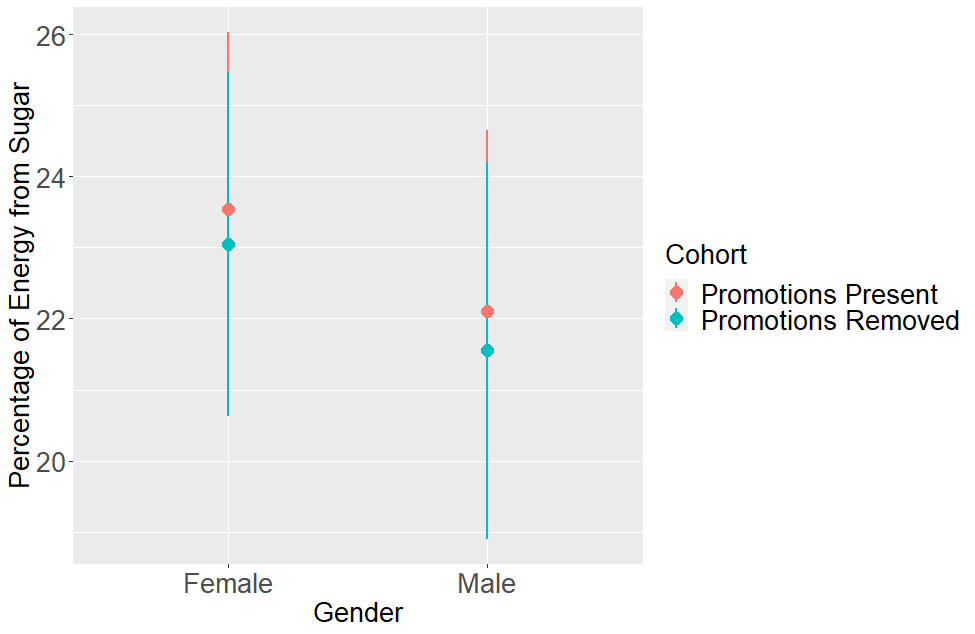 | 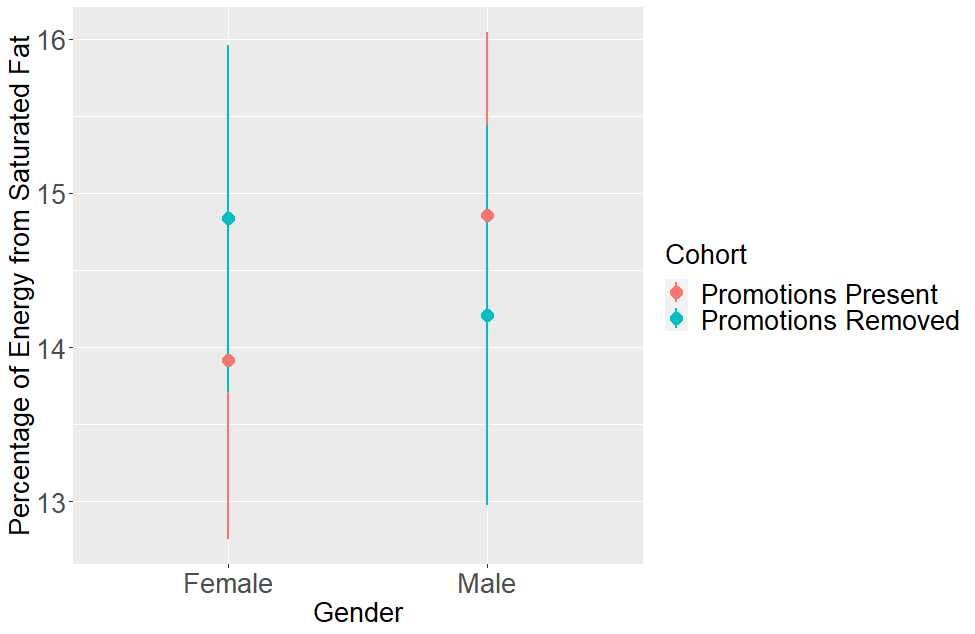 |
| 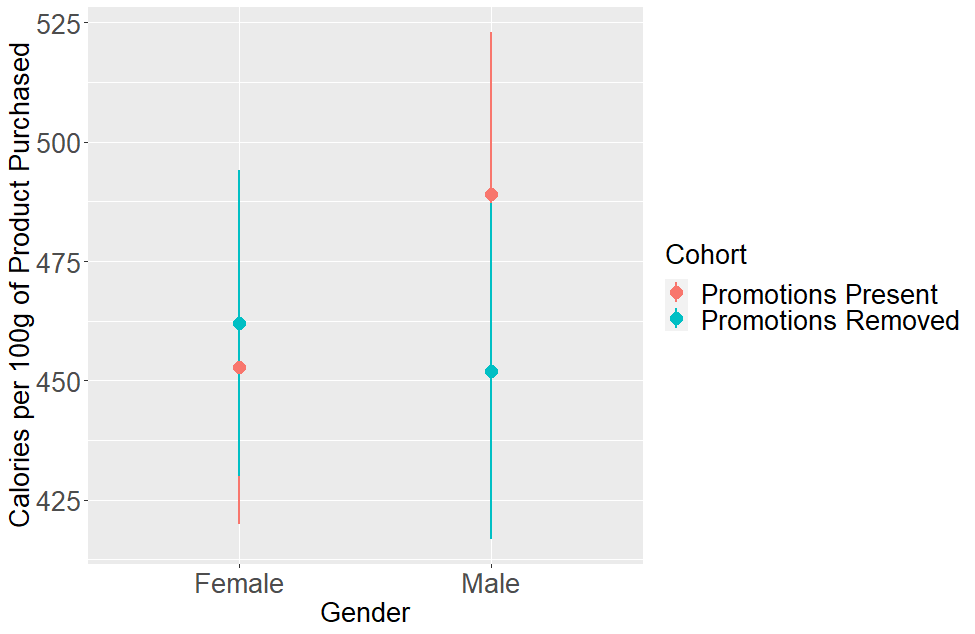 | |

## **Figure S2**. Marginalised mean plots for secondary outcomes by BMI groups

| 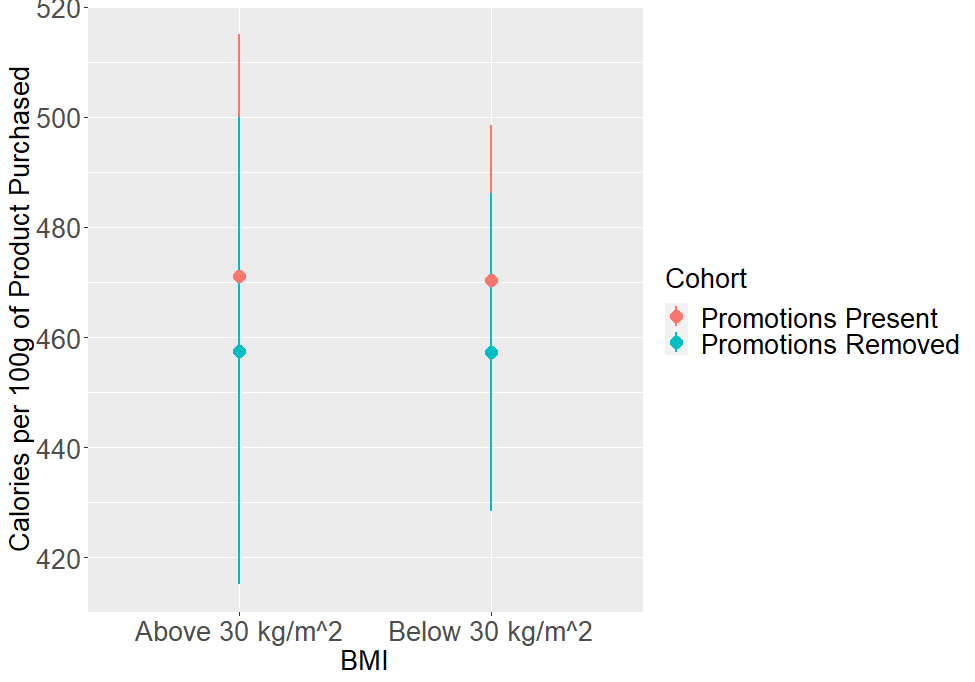 | 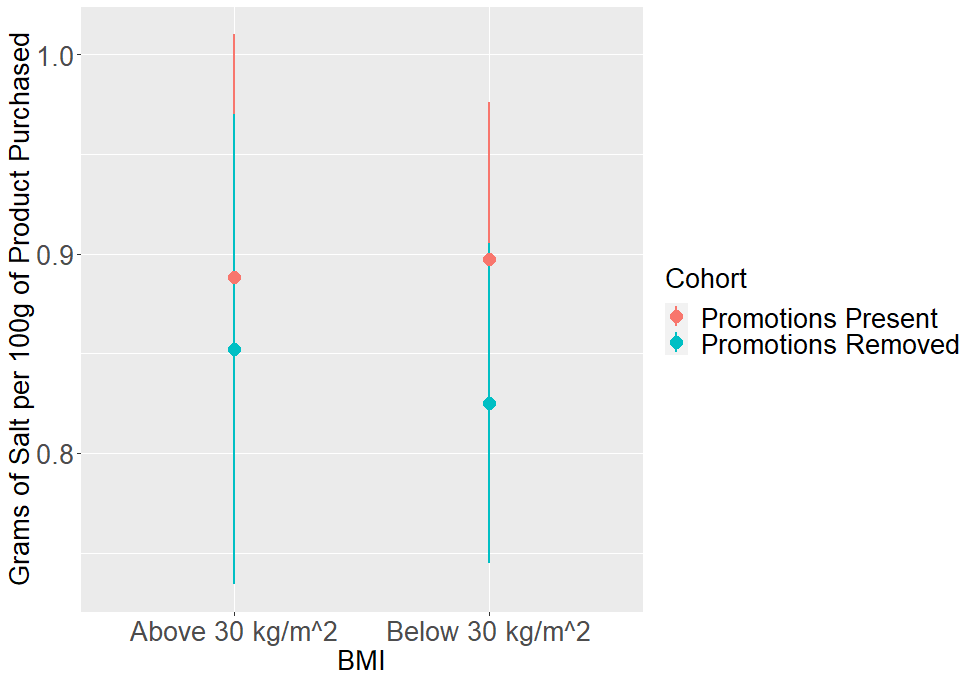 |
| --- | --- |
| 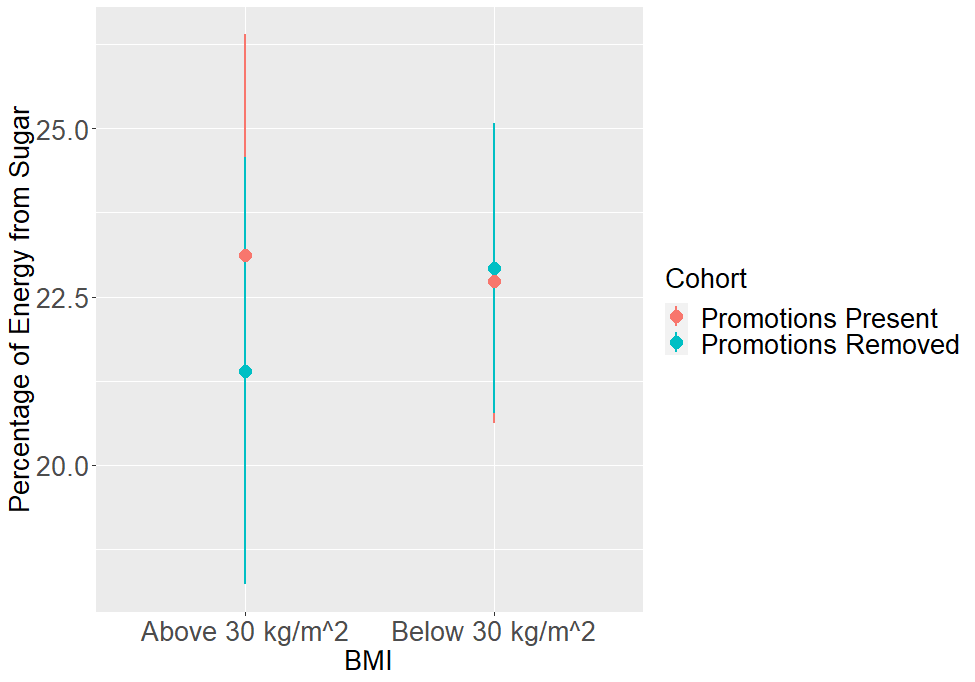 | 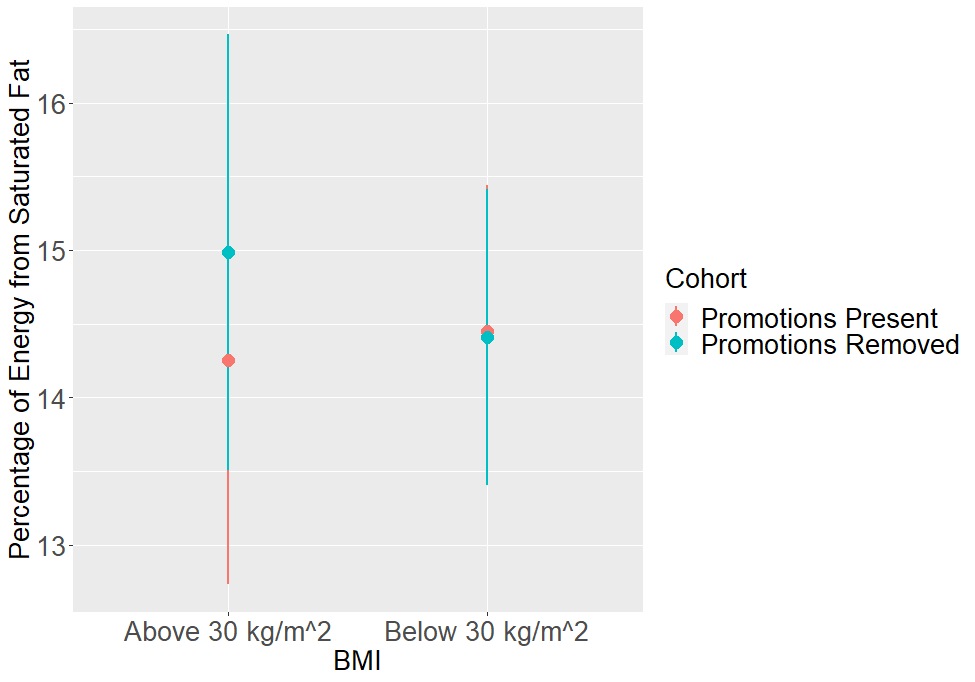 |
| 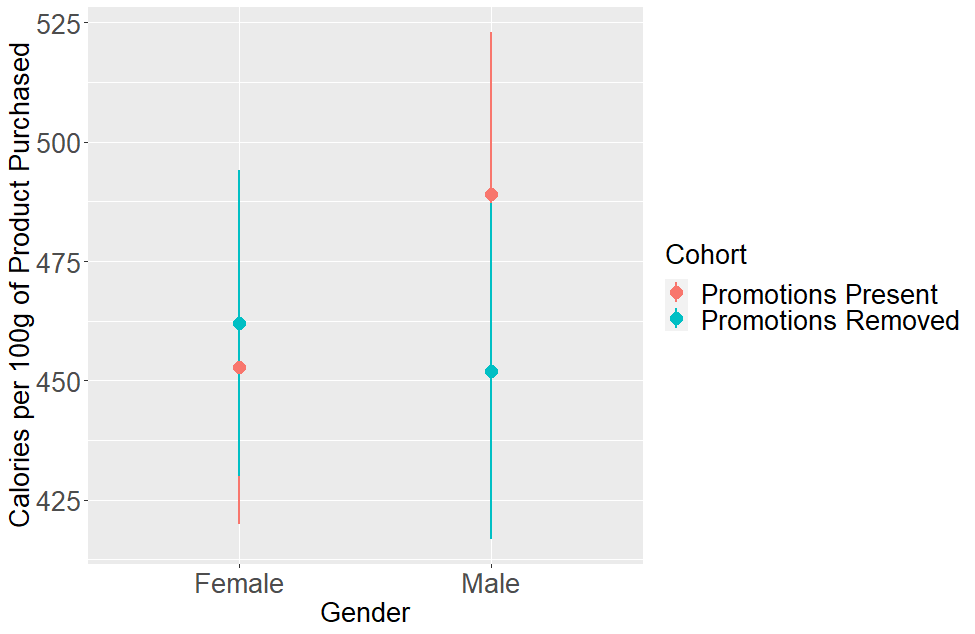 | |

## **Figure S3**. Marginalised mean plots for secondary outcomes by education level

| 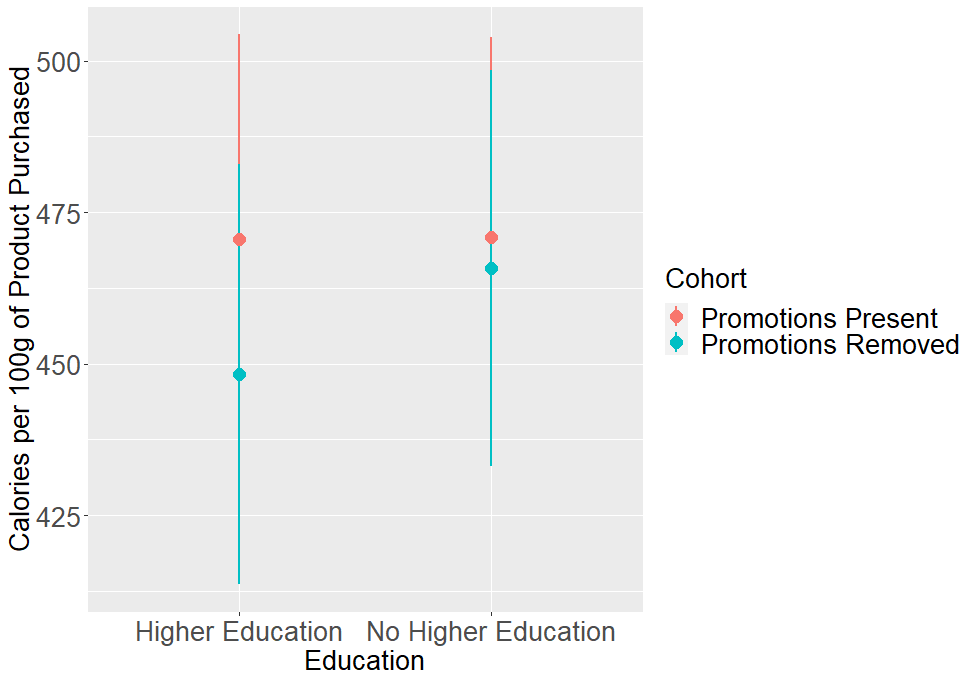 | 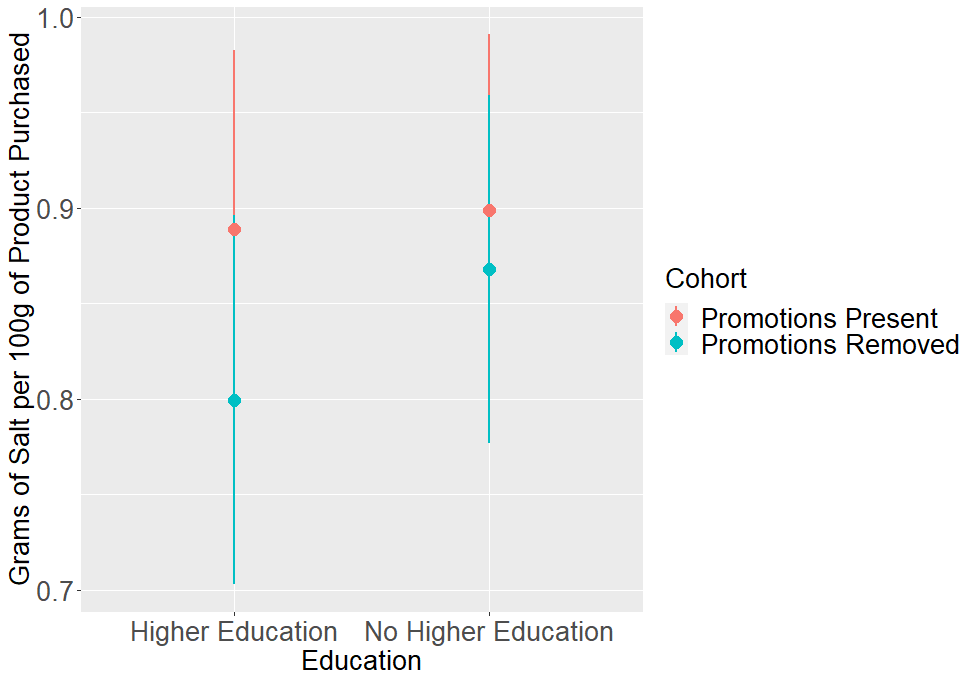 |
| --- | --- |
| 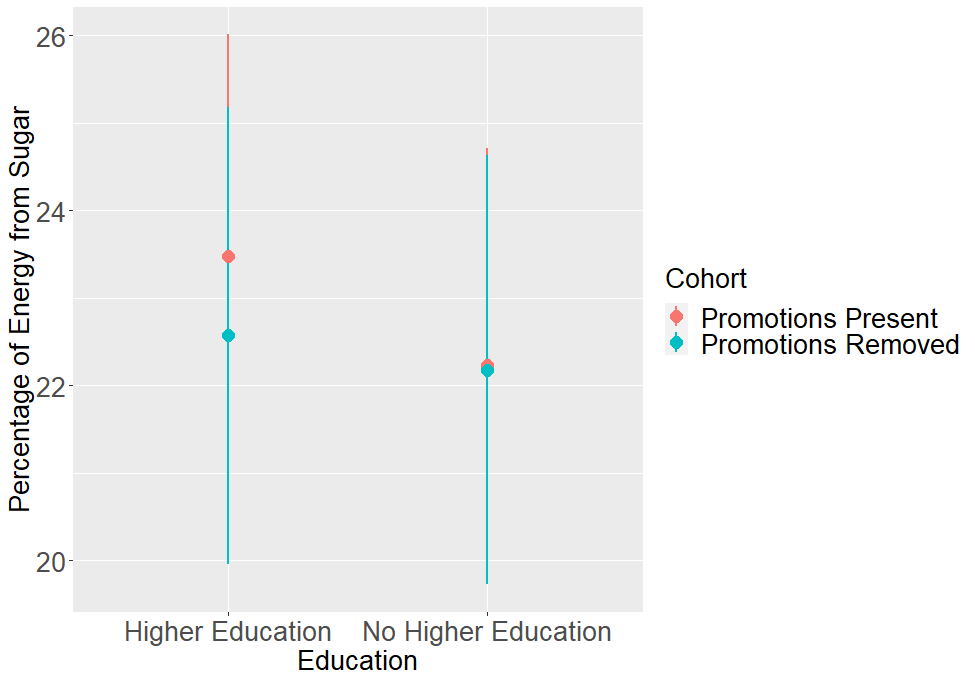 | 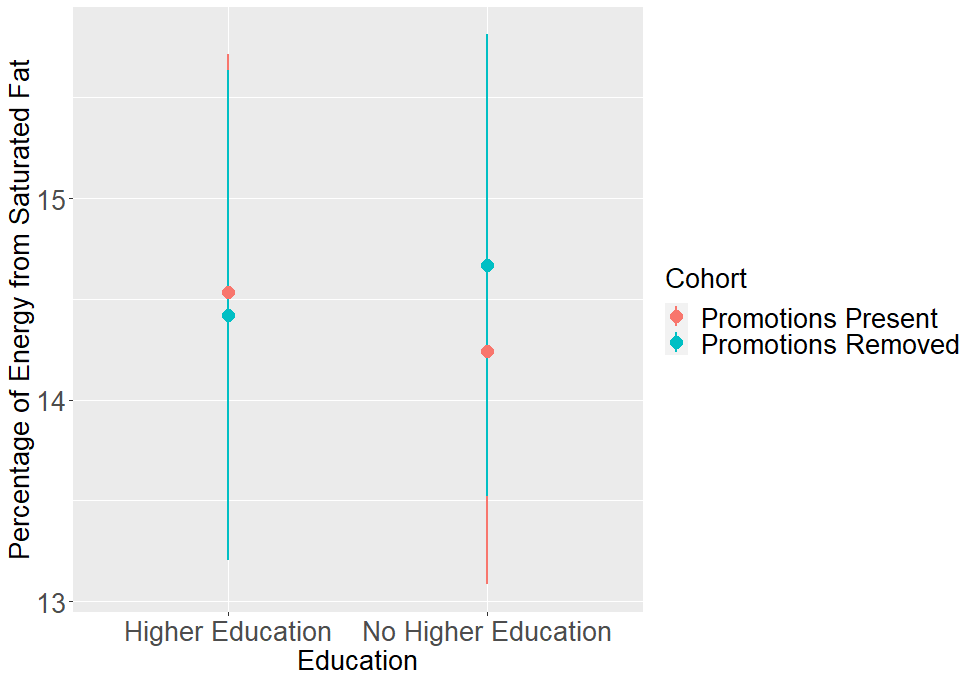 |
| 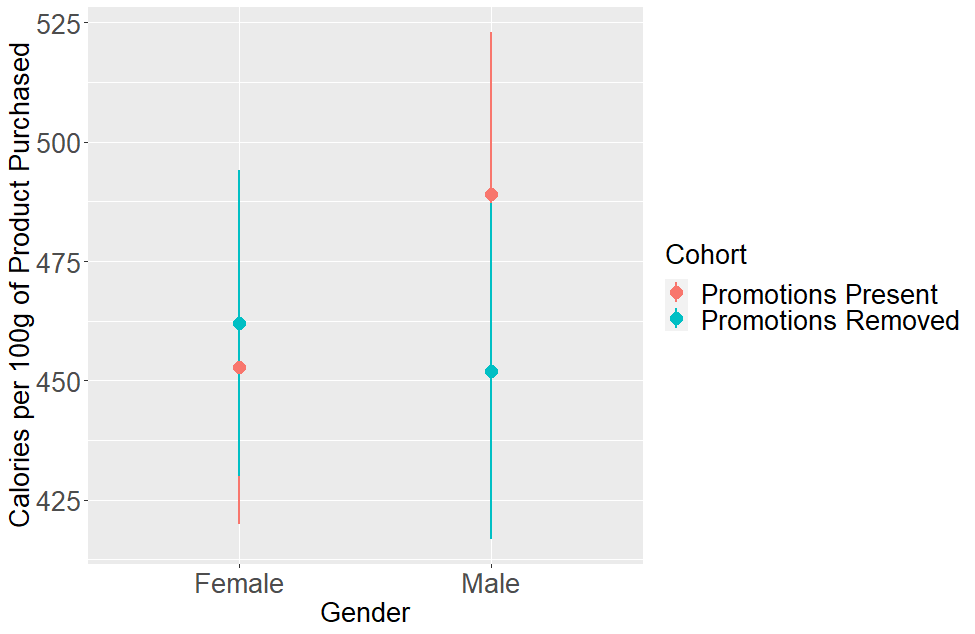 | |

## **Figure S4**. Marginalised mean plots for secondary outcomes by household income

| 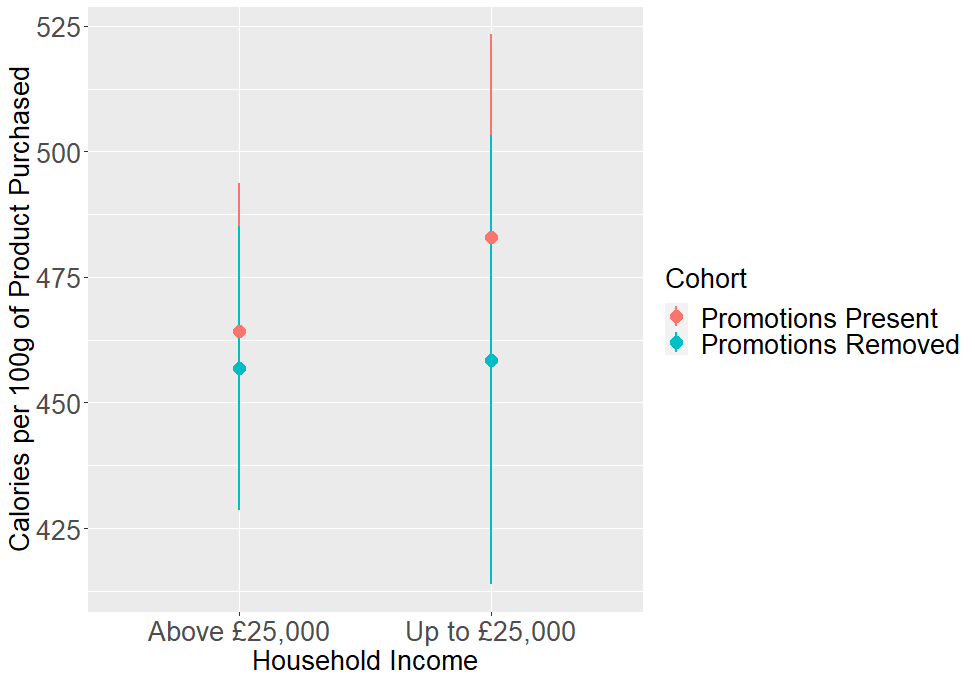 | 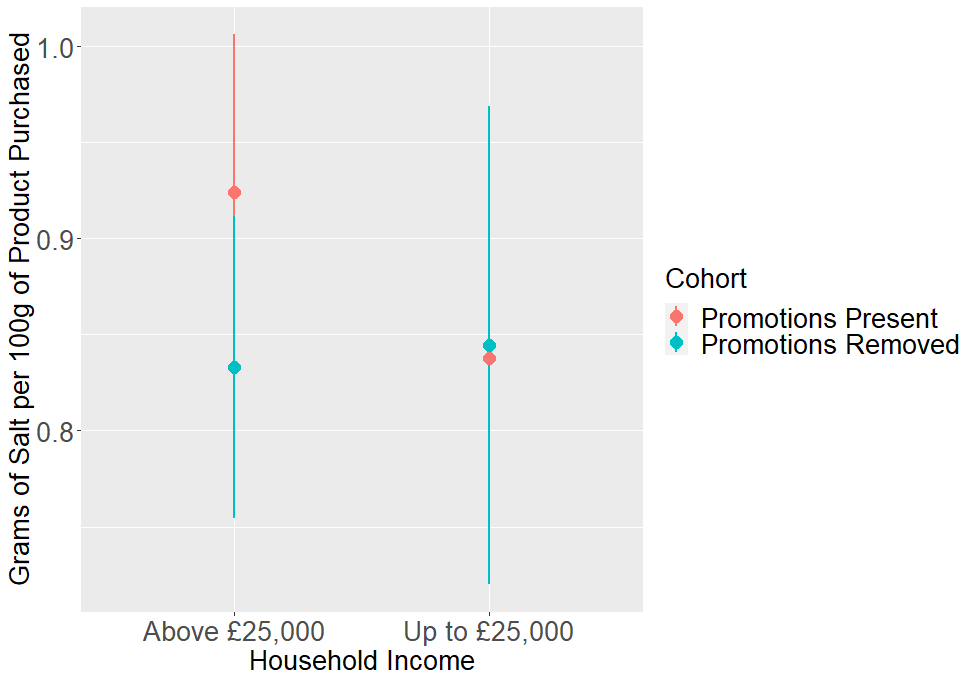 |
| --- | --- |
| 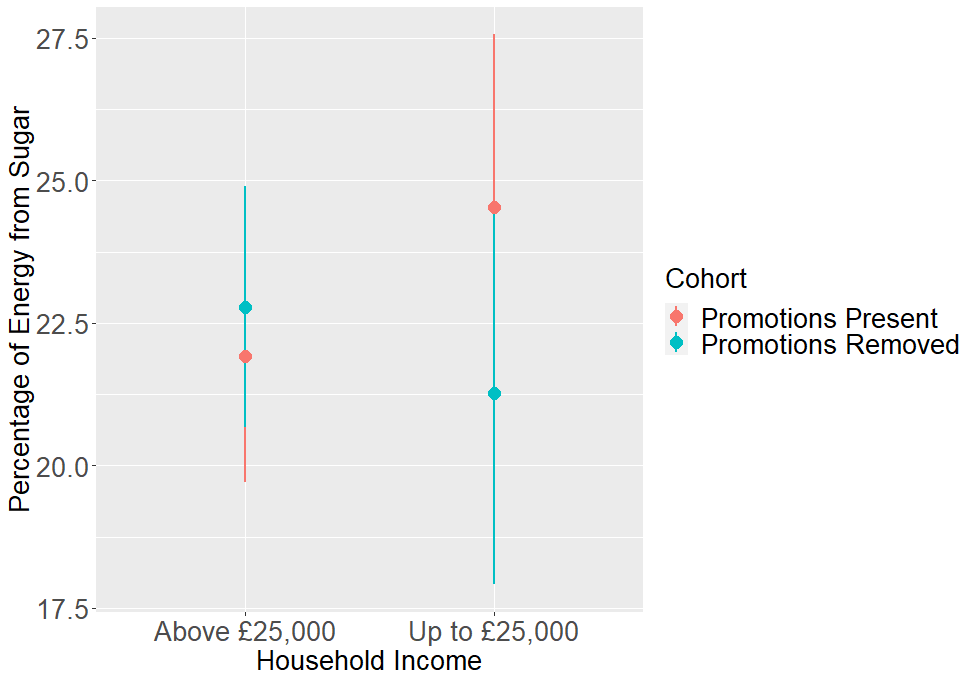 | 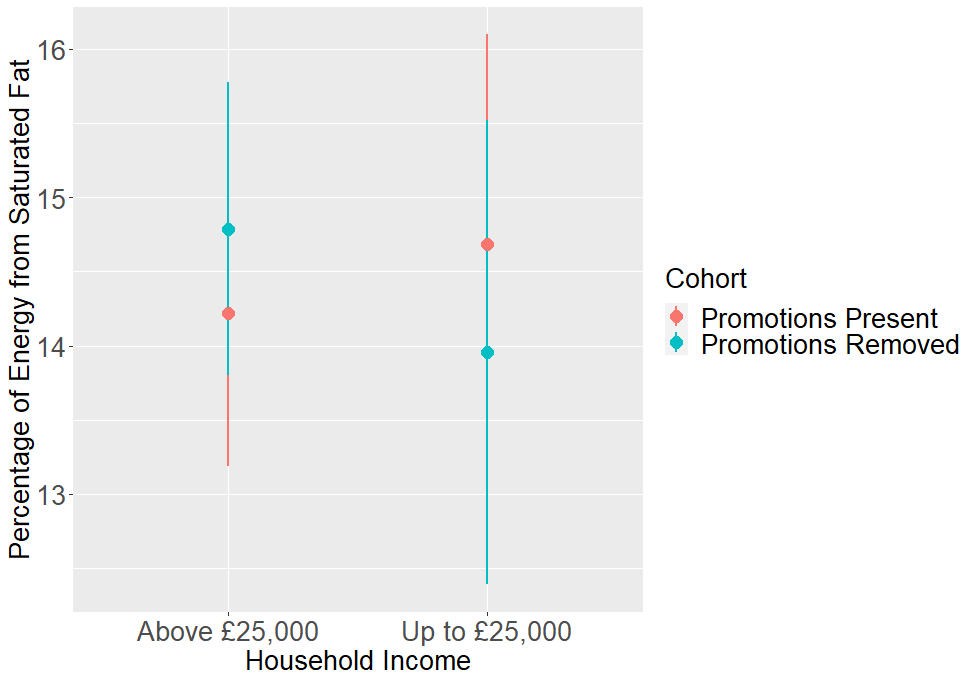 |
| 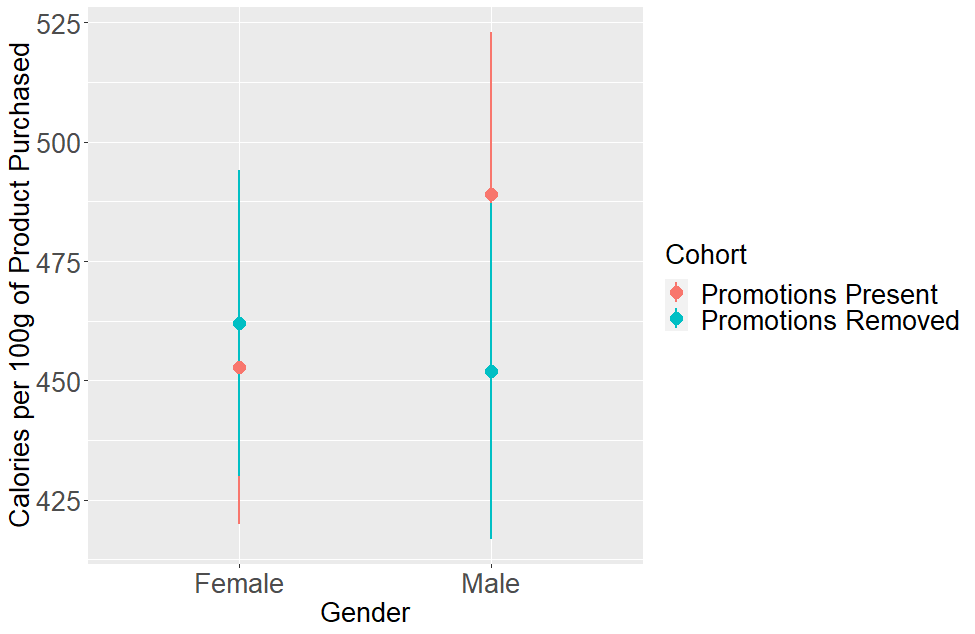 | |

## **Figure S5**. Marginalised mean plots for secondary outcomes by ethnicity group

| 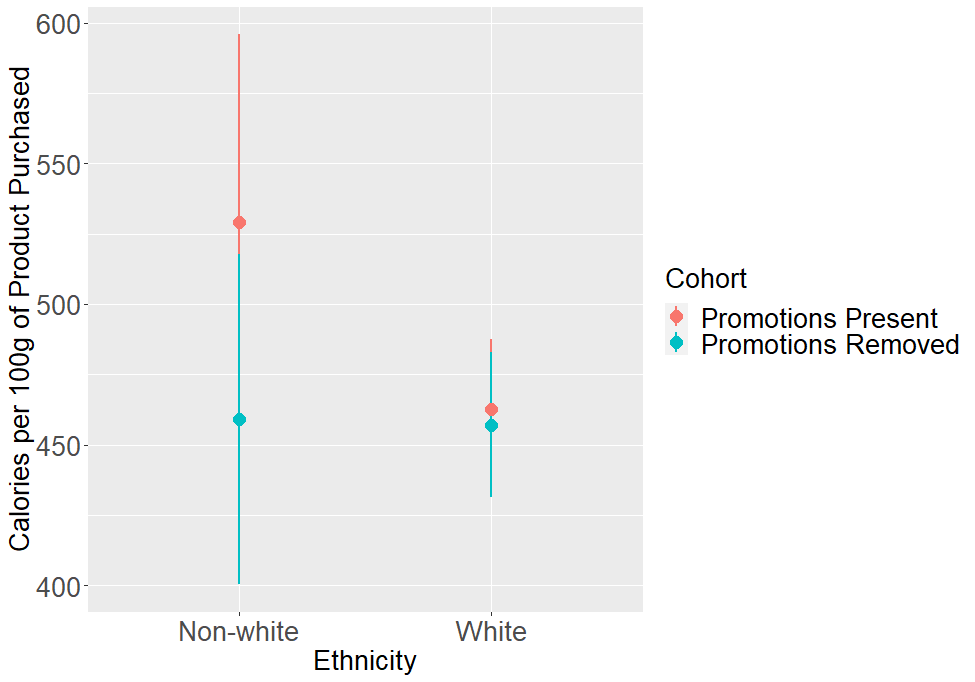 | 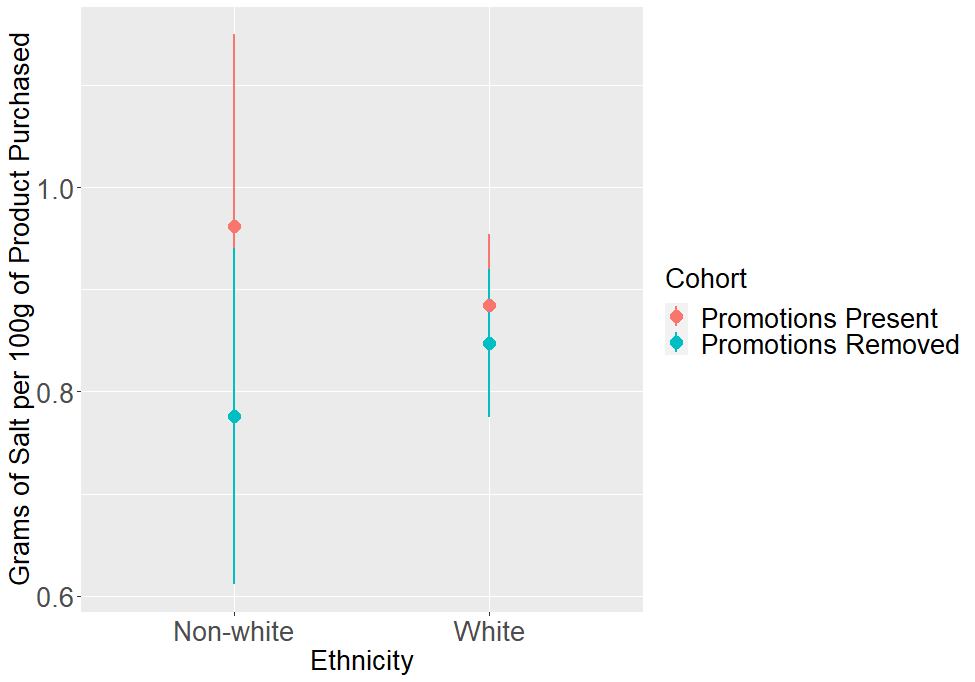 |
| --- | --- |
| 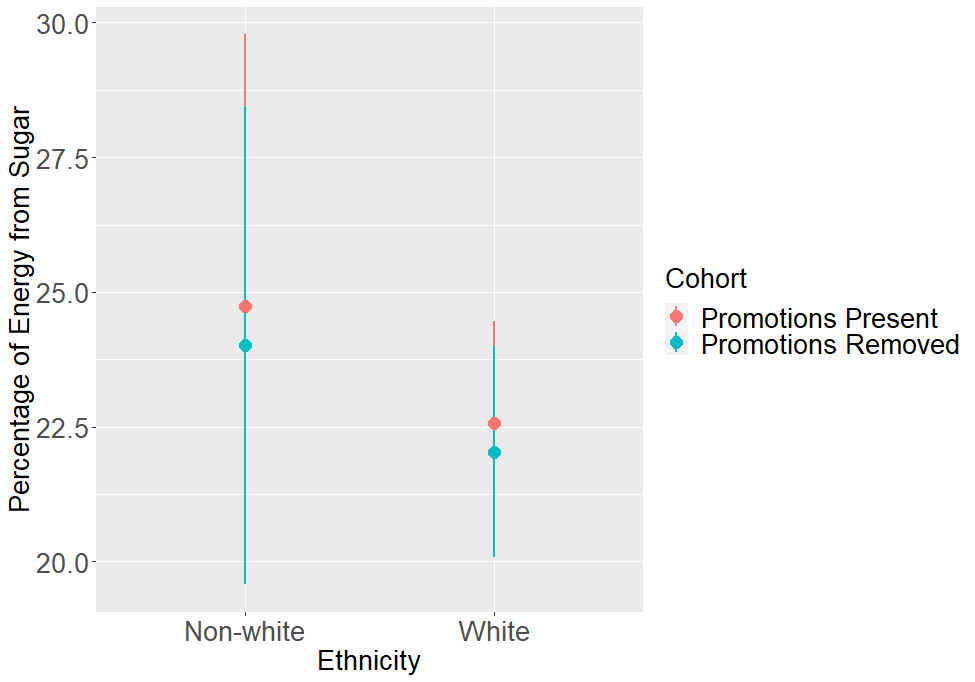 | 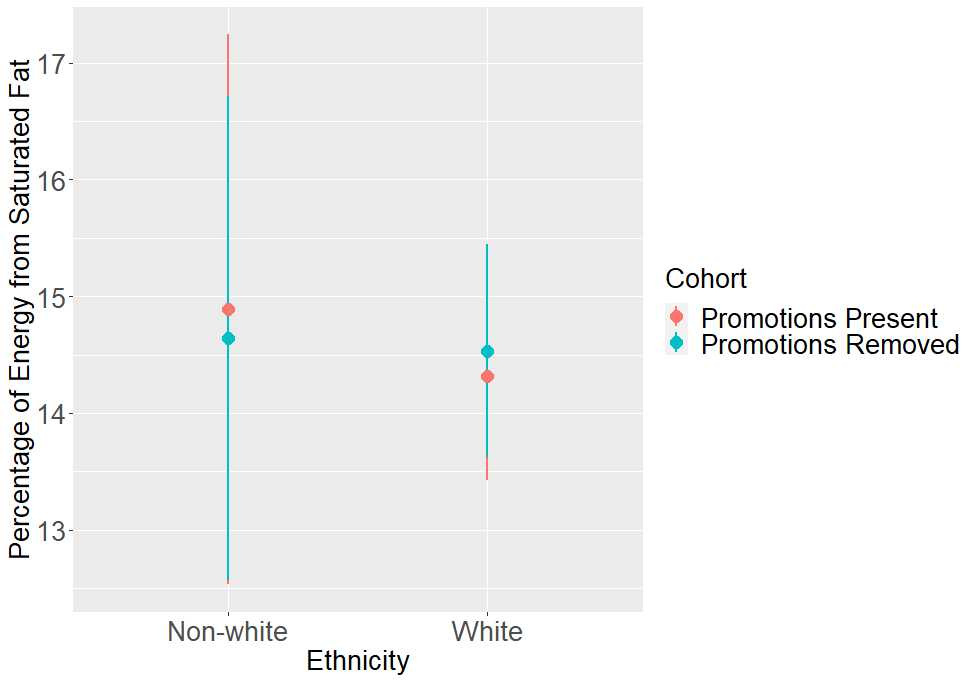 |
| 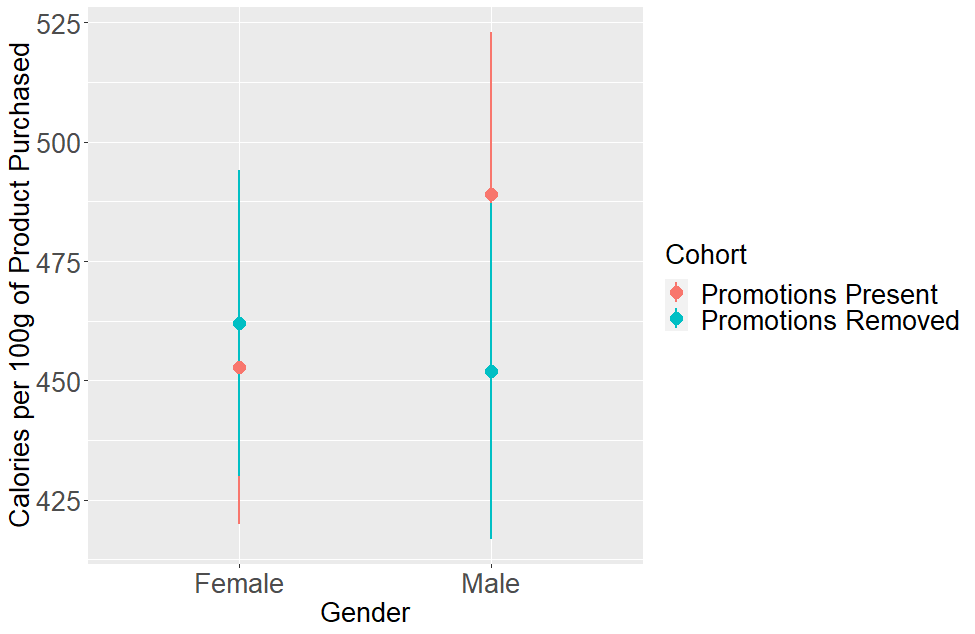 | |

## **Figure S6**. Marginalised mean plots for secondary outcomes by age group

| 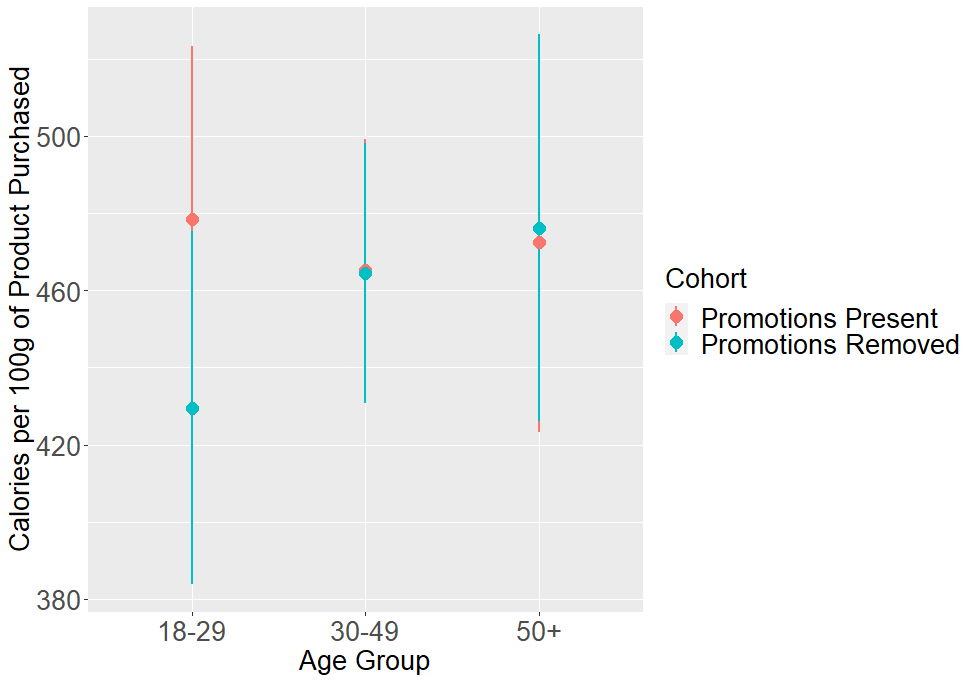 | 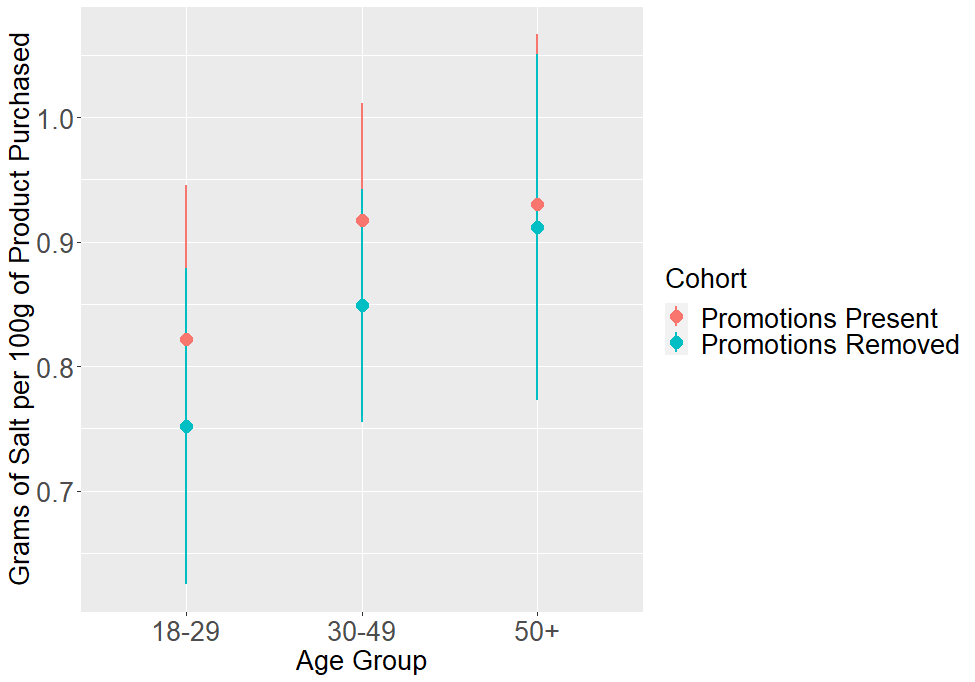 |
| --- | --- |
| 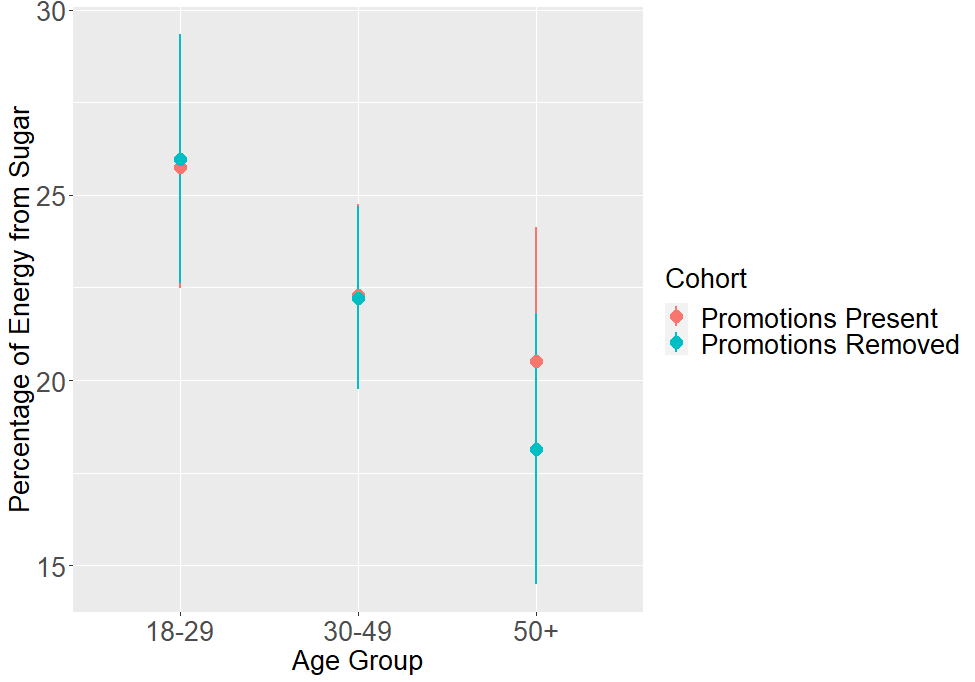 | 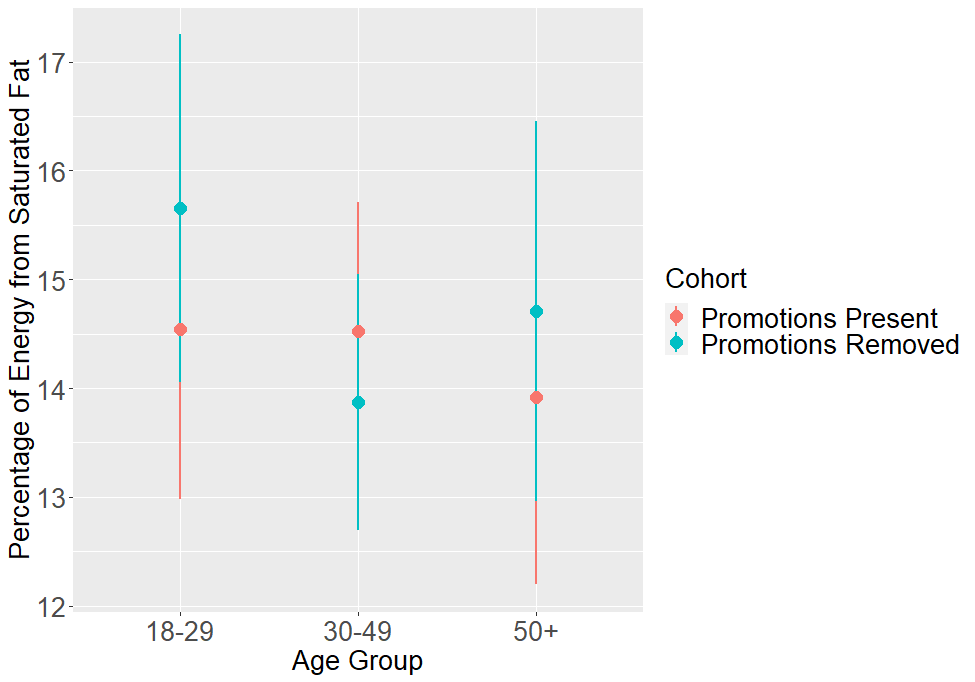 |
| 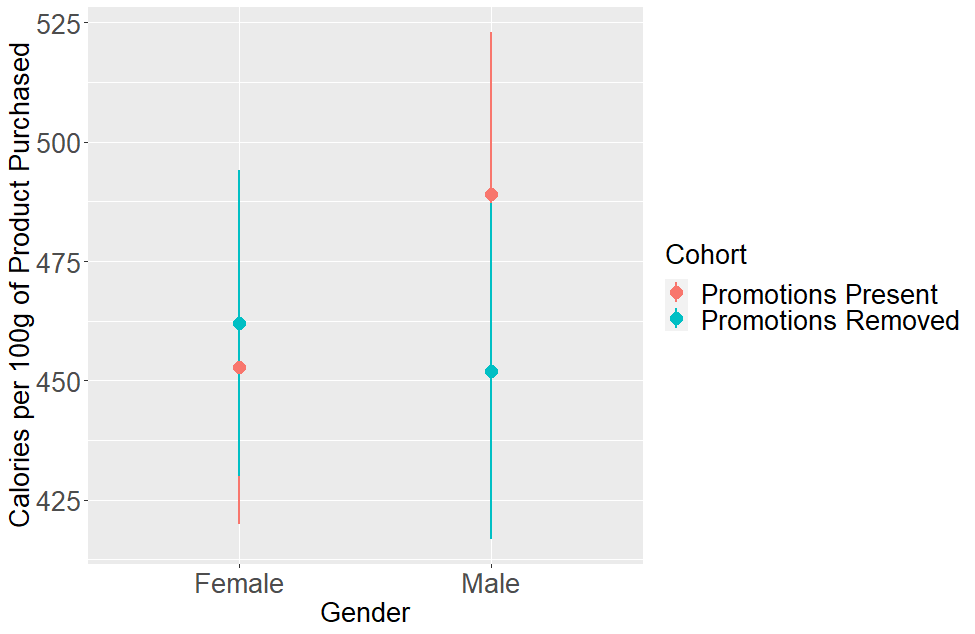 | |

## **Figure S7.** Example of the experimental supermarket with promotions.


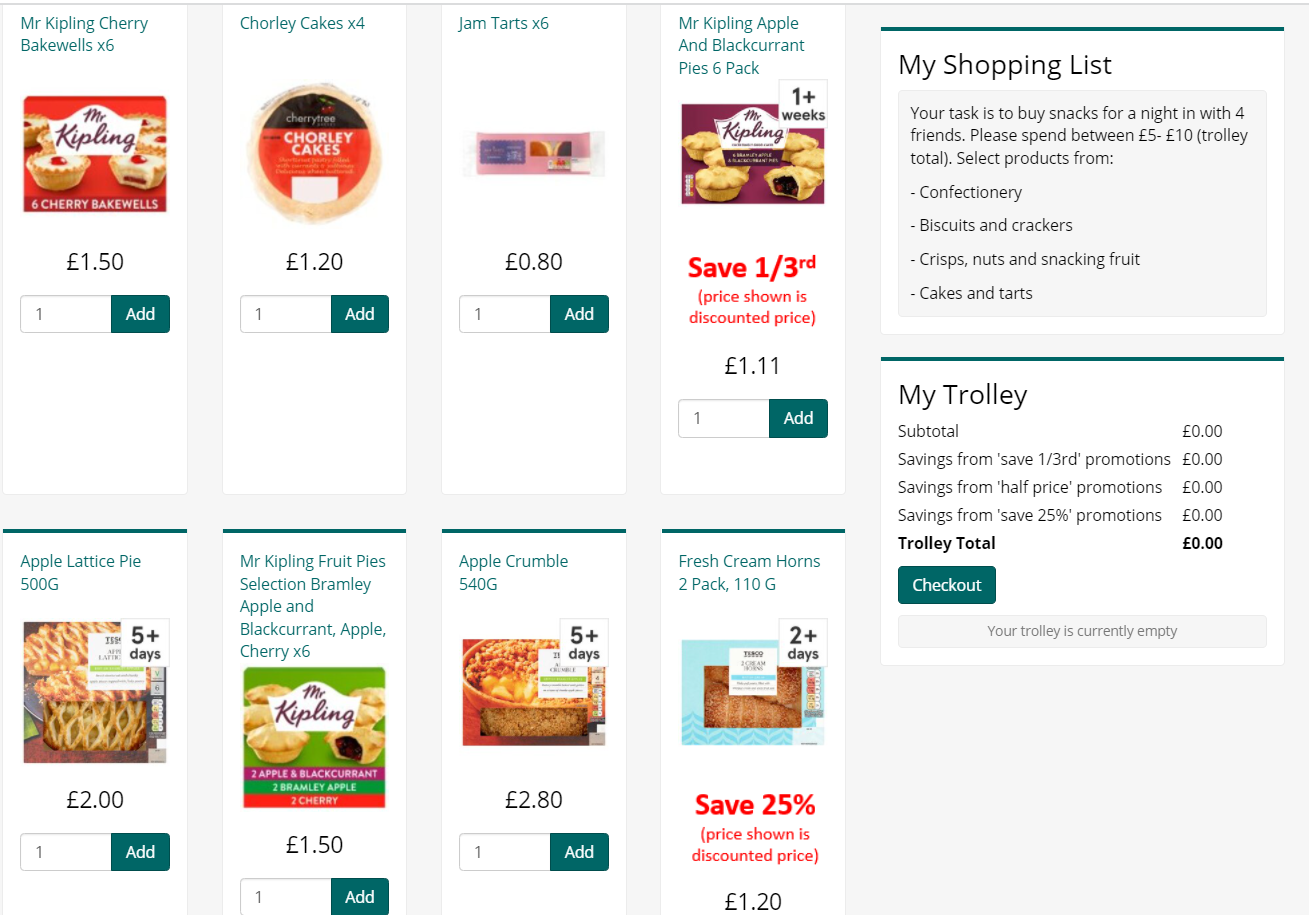


## **Figure S8.** Example of the experimental supermarket without promotions.


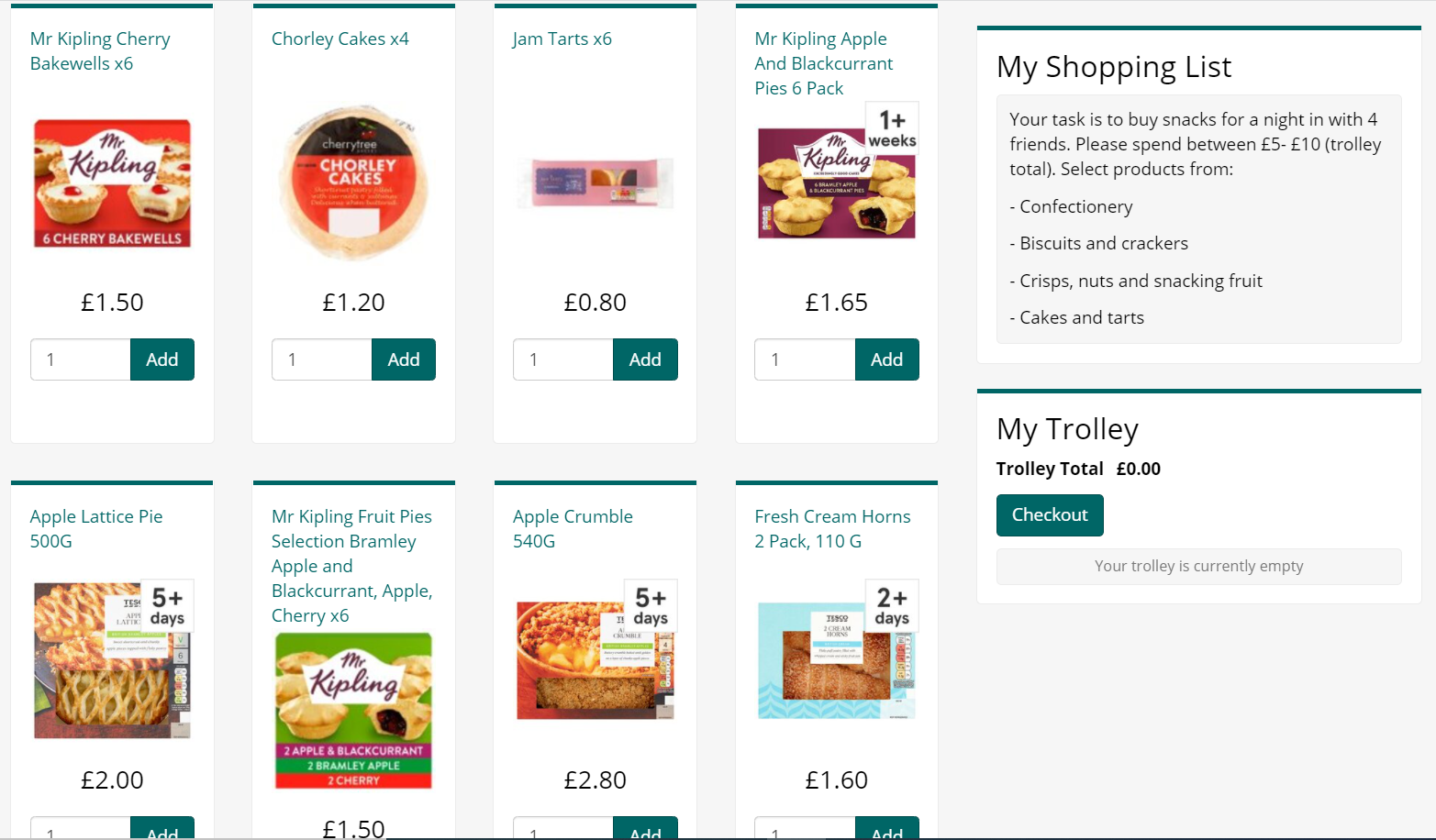

Supplement: Multimedia component 1 [file mmc1.docx]
